# Why women’s health financing collapses when donors retreat and interest payments rise

**DOI:** 10.3389/fgwh.2026.1894105

**Published:** 2026-10-21

**Authors:** Mayadhar Sethy

**Affiliations:** Department of Development Studies, Nabakrushna Choudhury Centre for Development Studies, Bhubaneswar, India

**Keywords:** debt service crowding-out, development assistance for health, government health expenditure, low- and lower-middle-income countries, sex-Disaggregated health spending, universal health coverage, women's health financing

## Abstract

**Background:**

Stagnant government health expenditure (GHE) and declining development assistance for health (DAH) threaten universal health coverage (UHC) for women in low-income countries (LICs) and lower-middle-income countries (LMICs). Women's health financing remains under-resourced and vulnerable to fiscal austerity, debt distress, and donor withdrawal. This study quantifies the crowding-out effect of debt service on government expenditure for women's health (GHEW) and estimates the probability of closing women-specific UHC financing gaps under alternative donor withdrawal scenarios.

**Methods:**

A balanced panel of 71 countries (32 LICs and 39 LMICs), comprising 497 country-years from 2018 to 2025, was constructed using WHO, IMF, World Bank, OECD, IHME, and DHS data. A fixed-effects instrumental-variable model estimated determinants of GHEW using lagged GHE and donor-alignment instruments (first-stage F = 41.2). A Cox survival model projected the probability of closing women's UHC financing gaps by 2030, defined as combined spending reaching ≥80% of sex-disaggregated benchmarks (US$62 for LICs and US$95 for LMICs). Projections incorporated announced donor reductions with probabilistic scenarios. Validation included out-of-sample forecasting, placebo tests, and patient-level DHS validation.

**Findings:**

Median combined LIC spending increased only marginally, from US$15.90 in 2018 to US$16.40 in 2025. Each 1-percentage-point increase in debt service as a share of GDP reduced GHEW by 4.2% (*β*=−0.042, *p* < 0.001), significantly exceeding its effect on general health expenditure (*β*=−0.012, *p* = 0.032; difference *p* = 0.008). Under baseline projections, no LIC and 23.1% of LMICs were projected to close women's UHC financing gaps by 2030. Each 1% decline in DAH reduced gap-closure probability by 6.1% (HR = 0.939, *p* = 0.002), whereas fiscal-space expansion nearly tripled closure likelihood (HR = 2.947, *p* = 0.018). Patient-level validation showed that a US$10 increase in predicted spending was associated with a 4.2-percentage-point increase in skilled birth attendance (95% CI 2.8–5.6).

**Interpretation:**

Debt service disproportionately crowds out women's health expenditure, highlighting gendered consequences of fiscal adjustment. Abrupt donor withdrawal makes UHC financing gaps virtually unattainable in aid-dependent LICs without domestic reprioritization. Debt-for-health swaps, phased donor transitions, and increased women's health expenditure shares are critical. Raising the women's health share by ≥2.5 percentage points could increase LIC gap-closure probability to approximately 31% by 2030 (range 22%–41%).

## 1 Introduction

### 1.1 Background and motivation: the gendered nature of health financing crises

The pursuit of Universal Health Coverage (UHC) remains one of the defining global health priorities of the twenty-first century, particularly within low-income countries (LICs) and lower-middle-income countries (LMICs), where financing systems continue to face structural fragility and widening inequalities. Although the Sustainable Development Goals (SDGs) established ambitious commitments to equitable health access by 2030, progress in health financing has slowed considerably during the post-2015 period, threatening the sustainability of health systems and disproportionately affecting women and girls (1, 2). Women remain more dependent on public health systems because of their greater reproductive, maternal, sexual, and caregiving health needs, yet financing for women's health services continues to lag behind required investment thresholds (3, 4). Consequently, the contemporary financing crisis is not gender neutral; rather, it reflects a deeply gendered political economy of public expenditure in which women bear the largest burden of fiscal austerity, underinvestment, and declining external assistance (5–7).

Women's health investment constitutes a central pillar of human capital formation and long-run economic development. Empirical evidence demonstrates that healthier women exhibit higher labour productivity, greater workforce participation, improved educational attainment, and stronger intergenerational welfare outcomes (1, 3). Investments in maternal health, reproductive services, family planning, and primary healthcare also generate substantial economic multipliers through reductions in mortality, improved child development, and enhanced household productivity (3, 8). The World Bank's Human Capital framework similarly identifies women's health as a prerequisite for sustainable economic growth and poverty reduction (2). Despite these well-established benefits, financing commitments remain critically inadequate across most LICs and LMICs. Recent estimates from the World Health Organization (WHO) indicate that the median LIC allocated only approximately US$16.40 per capita for women's health services in 2025, substantially below the minimum benchmark of roughly US$62 per capita required to achieve essential UHC targets for women's health (4, 5). Such persistent financing gaps reveal profound disparities between international commitments and actual resource mobilization.

Structural inequalities in health financing perpetuate gender disparities in health outcomes, with women in LICs and LMICs experiencing compounded vulnerabilities from both fiscal austerity and weak governance systems (38). Their analysis of health production functions reveals that government health expenditure has a stronger positive effect on women's health outcomes in countries with higher governance quality and gender-responsive budgeting mechanisms (39). Identify health system performance determinants across LMICs, highlighting that debt service burdens significantly undermine the capacity of health systems to deliver equitable care for women and children (40), extend this analysis by examining the gendered dimensions of nutrition and food security, revealing how fiscal constraints disproportionately affect women's nutritional status through reduced public health spending.

The financing crisis affecting women's health systems is driven by multiple intersecting pressures. First, macroeconomic instability has constrained fiscal space across developing economies. Weak economic growth, elevated debt burdens, rising debt-service obligations, and limited domestic revenue mobilization have significantly reduced the capacity of governments to expand public health expenditure (6, 9). Fiscal consolidation programmes implemented in response to debt distress frequently prioritize expenditure compression over social investment, leading to disproportionate reductions in sectors associated with social reproduction and care work, including maternal and reproductive health services (7, 10). The IMF Fiscal Monitor and related fiscal space literature demonstrate that debt servicing increasingly crowds out developmental expenditures in LICs, thereby limiting governments’ ability to finance essential health services (6, 20, 24, 34, 41) further demonstrate that debt service burdens in South Asian countries are associated with reduced government spending on nutrition and maternal health programmes, with the poorest and most marginalized women bearing the greatest burden of these fiscal adjustments.

Second, women's health often receives lower political priority within national health budgets despite its broad developmental importance. Gender-blind budgeting structures continue to dominate public financial management systems, preventing governments from identifying the differentiated impacts of health expenditure on women and men (12, 13). Studies on gender-responsive budgeting reveal that expenditure allocation processes frequently overlook women's specific healthcare needs, particularly in reproductive health, antenatal care, menstrual health, and gender-based violence services (13, 16, 17). Evidence from cross-country analyses further indicates that women's health expenditure shares have declined in several LMICs during the SDG period, reflecting institutional weaknesses in gender-sensitive fiscal planning (8, 16). Theoretical frameworks developed by Elson and Stotsky argue that gender-responsive budgeting is essential for correcting structural biases in fiscal allocation and improving distributive equity in health systems (12, 13, 42) provide empirical evidence from India showing that gender-responsive budgeting mechanisms significantly improve women's access to health services, particularly in states with stronger institutional capacity and civil society engagement further demonstrate that primary healthcare investments generate the highest returns for women's health outcomes, particularly when combined with targeted nutrition interventions and community-based health programmes (43).

Third, and perhaps most critically, declining development assistance for health (DAH) threatens to reverse decades of progress in women's health financing. External assistance has historically played a major role in financing maternal health, HIV/AIDS programmes, sexual and reproductive health services, and family planning interventions in LICs and fragile states (21, 22). However, recent reductions in donor commitments have generated severe uncertainty regarding the sustainability of health financing systems (9–11). OECD projections indicate that official development assistance experienced its largest contraction in recorded history in 2025, while major bilateral donors—including the United States, the United Kingdom, and France—announced significant reductions in aid allocations (9–11). These cuts are expected to disproportionately affect women-focused programmes, particularly reproductive health and HIV/AIDS services, which rely heavily on donor financing (10, 28–31). Existing evidence also suggests that reductions in external assistance are not always compensated by equivalent increases in domestic public expenditure, thereby increasing the likelihood of widening financing gaps (23).

The broader literature on health financing demonstrates that sustained public expenditure remains essential for achieving UHC outcomes. Research by Reeves et al. highlights the importance of progressive taxation systems in strengthening public health financing capacity across LMICs (14). Similarly, Xu et al. identify minimum health expenditure thresholds necessary to protect populations against catastrophic health expenditure and financial risk (15). Tandon and Cashin conceptualize fiscal space for health as the government's capacity to increase spending without undermining macroeconomic sustainability, while Kutzin emphasizes that effective health financing systems must simultaneously improve equity, efficiency, and financial protection (24, 25). Recent modelling exercises from Disease Control Priorities and the Lancet Global Health Commission further estimate that achieving essential UHC packages will require substantial increases in domestic health expenditure, especially within primary healthcare systems serving women and vulnerable populations (26, 27).

Against this background, this study investigates the determinants of government health expenditure trajectories for women's health services across 71 LICs and LMICs between 2018 and 2030. The paper contributes to the literature in five major ways. First, it develops an integrated panel econometric framework linking macro-fiscal conditions, institutional governance, external assistance, and women-specific UHC financing outcomes. Second, it applies survival analysis techniques to estimate the probability of countries achieving minimum financing thresholds for women's health services by 2030. Third, it incorporates GIS-based spatial analysis to identify regional clustering and geographic concentration of financing gaps. Fourth, the study strengthens methodological reliability through multi-database validation, out-of-sample forecasting, falsification tests, and robustness checks across alternative specifications. Finally, it provides policy simulations evaluating the impact of aid reductions, fiscal expansion, and gender-responsive budgeting reforms on future women's health financing trajectories.

The conceptual framework underpinning this study integrates macro-fiscal theory, gender budgeting literature, and health financing models to explain the dynamics of women's health expenditure under conditions of fiscal constraint. Classical fiscal theories developed by Musgrave, Rostow, and Wagner emphasize the relationship between state capacity, economic development, and public expenditure expansion (35–37). Building upon these foundations, contemporary fiscal space frameworks suggest that GDP growth, domestic revenue mobilization, and debt sustainability jointly shape governments’ capacity to finance health systems (24, 34). Within this framework, gender-responsive budgeting operates as a moderating mechanism influencing expenditure allocation toward women's health priorities (12, 13, 17).

Figure 1 conceptualizes these relationships by illustrating how macroeconomic determinants influence fiscal space, which subsequently affects the allocation of government health expenditure for women's services (GHEW). Debt-service obligations compete directly with social spending, while external development assistance supplements domestic financing. Government expenditure for women's health combines with off-budget donor financing to determine total women's health investment levels. The framework also recognizes dynamic feedback effects whereby improvements in women's health contribute to labour productivity, demographic transitions, and long-term economic growth, thereby reinforcing future fiscal capacity (1–3, 33). In this sense, women's health financing should not be viewed merely as social expenditure but as a strategic investment in sustainable development and inclusive economic transformation.

**Figure 1:**
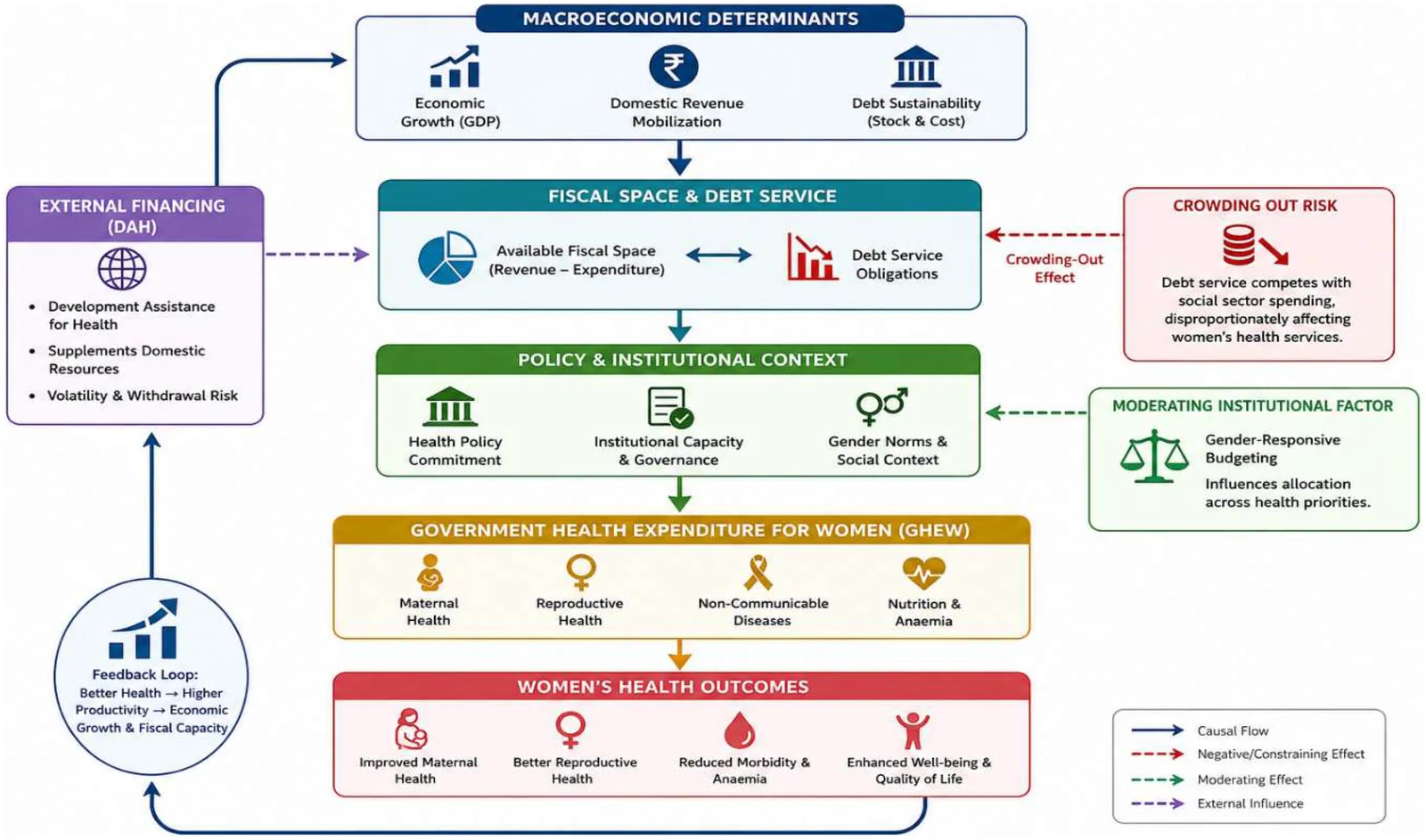
Conceptual framework for women's health financing under fiscal constraint. Source: Author's conceptualization.

Overall, the study situates women's health financing at the centre of debates surrounding fiscal sustainability, development assistance, and UHC achievement. As LICs and LMICs confront intensifying fiscal pressures during the SDG period, understanding the determinants of women's health expenditure becomes essential for designing equitable and resilient financing systems capable of preventing reversals in health and gender equality outcomes.

## 2 Methods

### 2.1 Data sources and multi-database validation strategy

This study draws on eight data sources spanning 2018–2030 for 71 countries (32 LICs and 39 LMICs). Government health expenditure (GHE) data for women's health services for 2018–2025 were compiled from: the WHO Global Health Expenditure Database (GHED); the WHO Global Health Observatory (GHO); the World Bank Gender Data Portal; the OECD Creditor Reporting System (CRS); the IMF World Economic Outlook (WEO); the IHME Development Assistance for Health Database; Demographic and Health Surveys (DHS); and country-specific budget documents (over 6,000 government financial documents, concluding April 30, 2025). For country-years with missing data (18 countries in 2023; 41 in 2024; 44 in 2025), nowcasting techniques were employed.

Table 1 presents data sources and coverage by country, and Table 2 provides summary statistics for women’s health financing indicators (2018–2025, pooled sample, *n* = 497 country-years).

**Table 1:** Data sources and coverage by country, 2018–2025.

Country	Income group	Region	Years with GHE women's health	Years with DAH women's health	Years with budget data	DHS data available	Gender budgeting
Afghanistan	LIC	South Asia	2018–2024	2018–2024	2018–2023	2015	No
Angola	LMIC	Sub-Saharan Africa	2018–2025	2018–2024	2018–2025	2016	No
Bangladesh	LMIC	South Asia	2018–2025	2018–2025	2018–2025	2017, 2022	Yes (2015)
Benin	LIC	Sub-Saharan Africa	2018–2025	2018–2025	2018–2024	2017, 2021	No
Bhutan	LMIC	South Asia	2018–2025	2018–2023	2018–2025	2010	Yes (2012)
Bolivia	LMIC	Latin America	2018–2025	2018–2024	2018–2025	2008	Yes (2011)
Burkina Faso	LIC	Sub-Saharan Africa	2018–2025	2018–2025	2018–2024	2010, 2021	No
Burundi	LIC	Sub-Saharan Africa	2018–2024	2018–2024	2018–2023	2016, 2020	No
Cabo Verde	LMIC	Sub-Saharan Africa	2018–2025	2018–2023	2018–2025	2005	Yes (2014)
Cambodia	LMIC	East Asia	2018–2025	2018–2025	2018–2025	2014, 2021	Yes (2013)
Cameroon	LMIC	Sub-Saharan Africa	2018–2025	2018–2025	2018–2024	2011, 2018	No
Central African Republic	LIC	Sub-Saharan Africa	2018–2024	2018–2024	2018–2023	2019	No
Chad	LIC	Sub-Saharan Africa	2018–2024	2018–2024	2018–2023	2014, 2019	No
Comoros	LMIC	Sub-Saharan Africa	2018–2024	2018–2023	2018–2023	2012	No
Congo Dem. Rep.	LIC	Sub-Saharan Africa	2018–2025	2018–2025	2018–2024	2013, 2020	No
Congo Rep.	LMIC	Sub-Saharan Africa	2018–2024	2018–2024	2018–2023	2011	No
Côte d'Ivoire	LMIC	Sub-Saharan Africa	2018–2025	2018–2025	2018–2024	2011, 2021	Yes (2016)
Djibouti	LMIC	Sub-Saharan Africa	2018–2024	2018–2023	2018–2023	2006	No
Egypt	LMIC	Middle East	2018–2025	2018–2025	2018–2025	2014, 2021	Yes (2010)
El Salvador	LMIC	Latin America	2018–2025	2018–2024	2018–2025	2008	Yes (2012)
Eritrea	LIC	Sub-Saharan Africa	2018–2023	2018–2023	2018–2022	2010	No
Eswatini	LMIC	Sub-Saharan Africa	2018–2025	2018–2025	2018–2024	2006, 2021	No
Ethiopia	LIC	Sub-Saharan Africa	2018–2025	2018–2025	2018–2025	2016, 2019	Yes (2015)
Gambia	LIC	Sub-Saharan Africa	2018–2024	2018–2024	2018–2023	2013, 2019	No
Ghana	LMIC	Sub-Saharan Africa	2018–2025	2018–2025	2018–2025	2014, 2022	Yes (2011)
Guinea	LIC	Sub-Saharan Africa	2018–2024	2018–2024	2018–2023	2018	No
Guinea-Bissau	LIC	Sub-Saharan Africa	2018–2024	2018–2023	2018–2023	2014	No
Haiti	LIC	Latin America	2018–2024	2018–2024	2018–2023	2016, 2021	No
Honduras	LMIC	Latin America	2018–2025	2018–2024	2018–2025	2011, 2019	Yes (2013)
India	LMIC	South Asia	2018–2025	2018–2025	2018–2025	2015, 2019, 2021	Yes (2007)
Indonesia	LMIC	East Asia	2018–2025	2018–2025	2018–2025	2017, 2021	Yes (2010)
Kenya	LMIC	Sub-Saharan Africa	2018–2025	2018–2025	2018–2025	2014, 2022	Yes (2011)
Kiribati	LMIC	Pacific	2018–2024	2018–2023	2018–2023	2009	No
Kyrgyzstan	LMIC	Central Asia	2018–2025	2018–2024	2018–2025	2012, 2021	Yes (2012)
Lao PDR	LMIC	East Asia	2018–2025	2018–2024	2018–2024	2011, 2017	Yes (2014)
Lesotho	LMIC	Sub-Saharan Africa	2018–2025	2018–2025	2018–2024	2009, 2014	No
Liberia	LIC	Sub-Saharan Africa	2018–2025	2018–2025	2018–2024	2013, 2019	Yes (2012)
Madagascar	LIC	Sub-Saharan Africa	2018–2025	2018–2025	2018–2024	2021	No
Malawi	LIC	Sub-Saharan Africa	2018–2025	2018–2025	2018–2024	2015, 2020	Yes (2013)
Mali	LIC	Sub-Saharan Africa	2018–2024	2018–2024	2018–2023	2018	No
Marshall Islands	LMIC	Pacific	2018–2024	2018–2023	2018–2023	2007	No
Mauritania	LMIC	Sub-Saharan Africa	2018–2025	2018–2024	2018–2024	2019	No
Micronesia	LMIC	Pacific	2018–2024	2018–2023	2018–2023	2008	No
Mongolia	LMIC	East Asia	2018–2025	2018–2024	2018–2025	2013, 2019	Yes (2011)
Morocco	LMIC	Middle East	2018–2025	2018–2025	2018–2025	2018	Yes (2012)
Mozambique	LIC	Sub-Saharan Africa	2018–2025	2018–2025	2018–2024	2011, 2015, 2022	Yes (2014)
Myanmar	LMIC	East Asia	2018–2024	2018–2024	2018–2023	2015	No
Namibia	LMIC	Sub-Saharan Africa	2018–2025	2018–2025	2018–2025	2013	Yes (2010)
Nepal	LMIC	South Asia	2018–2025	2018–2025	2018–2025	2016, 2022	Yes (2011)
Nicaragua	LMIC	Latin America	2018–2024	2018–2024	2018–2023	2011	Yes (2012)
Niger	LIC	Sub-Saharan Africa	2018–2025	2018–2025	2018–2024	2012, 2021	No
Nigeria	LMIC	Sub-Saharan Africa	2018–2025	2018–2025	2018–2025	2018	Yes (2013)
Pakistan	LMIC	South Asia	2018–2025	2018–2025	2018–2025	2017, 2022	Yes (2009)
Papua New Guinea	LMIC	Pacific	2018–2024	2018–2024	2018–2023	2016	No
Philippines	LMIC	East Asia	2018–2025	2018–2025	2018–2025	2017, 2022	Yes (2010)
Rwanda	LIC	Sub-Saharan Africa	2018–2025	2018–2025	2018–2025	2014, 2019	Yes (2011)
Samoa	LMIC	Pacific	2018–2024	2018–2023	2018–2023	2014	No
São Tomé and Príncipe	LMIC	Sub-Saharan Africa	2018–2024	2018–2023	2018–2023	2008	No
Senegal	LMIC	Sub-Saharan Africa	2018–2025	2018–2025	2018–2024	2016, 2021	Yes (2012)
Sierra Leone	LIC	Sub-Saharan Africa	2018–2025	2018–2025	2018–2024	2019	No
Solomon Islands	LMIC	Pacific	2018–2024	2018–2023	2018–2023	2015	No
Somalia	LIC	Sub-Saharan Africa	2018–2023	2018–2023	2018–2022	2020	No
South Sudan	LIC	Sub-Saharan Africa	2018–2023	2018–2023	2018–2022	2010	No
Sri Lanka	LMIC	South Asia	2018–2025	2018–2025	2018–2025	2016	Yes (2010)
Sudan	LIC	Sub-Saharan Africa	2018–2024	2018–2024	2018–2023	2014	No
Tanzania	LIC	Sub-Saharan Africa	2018–2025	2018–2025	2018–2024	2015, 2022	Yes (2012)
Timor-Leste	LMIC	East Asia	2018–2025	2018–2024	2018–2024	2016	Yes (2013)
Togo	LIC	Sub-Saharan Africa	2018–2025	2018–2025	2018–2024	2013, 2021	No
Tuvalu	LMIC	Pacific	2018–2024	2018–2023	2018–2023	2007	No
Uganda	LIC	Sub-Saharan Africa	2018–2025	2018–2025	2018–2024	2016, 2021	Yes (2011)
Ukraine	LMIC	Europe	2018–2024	2018–2024	2018–2023	2007	Yes (2010)
Uzbekistan	LMIC	Central Asia	2018–2025	2018–2024	2018–2024	2006	Yes (2012)
Vanuatu	LMIC	Pacific	2018–2024	2018–2023	2018–2023	2013	No
Vietnam	LMIC	East Asia	2018–2025	2018–2025	2018–2025	2014, 2020	Yes (2011)
Yemen	LIC	Middle East	2018–2023	2018–2023	2018–2022	2013	No
Zambia	LMIC	Sub-Saharan Africa	2018–2025	2018–2025	2018–2024	2018	Yes (2012)
Zimbabwe	LMIC	Sub-Saharan Africa	2018–2025	2018–2025	2018–2024	2015	Yes (2013)

Years indicate data availability. Gender budgeting indicates existence of gender-responsive budgeting mechanisms in health sector (year of implementation in parentheses). *n* = 71 countries.

LIC, low-income country; LMIC, lower-middle-income country.

**Table 2:** Summary statistics for women's health financing indicators, 2018–2025 (pooled sample, *n* = 497 country-years).

Variable	Mean	Median	SD	Min	Max	Source	Obs.
GHE women's health per capita (2024 US$)	32.47	18.35	42.18	2.10	241.30	WHO GHED + budget docs	497
Off-budget DAH women's health per capita	9.87	5.24	15.63	0.00	98.70	OECD CRS + IHME	497
Women's health share of GHE (%)	16.82	15.70	7.45	4.20	42.10	Author calculation	497
Maternal health spending per capita (US$)	12.45	7.80	14.23	0.80	89.40	WHO + budget docs	497
Family planning spending per capita (US$)	4.23	2.10	5.67	0.10	34.20	WHO + FP2030	497
Reproductive cancer screening per capita (US$)	1.89	0.90	2.45	0.00	18.30	WHO + budget docs	485
Health share of GGE (%)	6.81	6.20	3.35	1.80	18.40	IMF WEO	497
GDP per capita (2024 US$)	1,892	1,543	1,465	210	4,950	IMF WEO	497
Debt service (% GDP)	4.23	3.80	3.12	0.20	18.70	IMF WEO	497
Tax revenue (% GDP)	13.42	12.80	5.23	4.50	28.90	IMF WEO	497
Gender budgeting index (0–100)	34.80	32.00	28.40	0.00	92.00	World Bank Gender Data	497
Maternal mortality ratio (per 100,000)	342	298	245	12	1,150	WHO GHO	497
Skilled birth attendance (%)	68.40	72.00	24.50	15.00	99.00	WHO GHO	497
Modern contraceptive prevalence (%)	35.20	32.00	18.40	5.00	82.00	WHO GHO	497
UHC women's health financing gap (2024 US$)	61.38	55.20	43.18	−28.40	98.50	Author Calculation	497

All spending in constant 2024 US dollars. Women's health includes maternal health, family planning, reproductive cancer screening, and other reproductive health services. Negative gap values indicate spending above benchmark (US 62LIC,US 95 LMIC).

GHE, government health expenditure; DAH, development assistance for health; GGE, general government expenditure; UHC, universal health coverage.

### 2.2 Outcome variables: women's health-specific measures

Primary outcome 1 was government health expenditure for women’s health per capita, estimated as (Equation 1):

$$\mathrm{GHE}W_{\mathrm{it}}=\mathrm{CGH}S_{\mathrm{it}}\times \omega_{\mathrm{it}}+\mathrm{SN}G_{\mathrm{it}}\times \omega_{\mathrm{it}}^{\mathrm{SNG}}+\mathrm{SH}I_{\mathrm{it}}\times \omega_{\mathrm{it}}^{\mathrm{SHI}}$$ (1)

where CGHS represents central government health spending for sexual and reproductive health and other women's health services, SNG denotes subnational spending weighted by women's health expenditure shares, and SHI refers to social health insurance contributions allocated to women's health services, including on-budget aid. The parameter *ω* captures the women's health share of spending derived from national health accounts and budget line-item allocations. For countries without sex-disaggregated health accounts (*n* = 34), women's health expenditure was estimated using service-specific expenditure tracking based on WHO SHA 2011 framework categories HC.1 (curative care by sex) and HC.5 (preventive care including maternal and reproductive health).

Primary outcome 2 measured the UHC financing gap for women’s health services and was estimated as (Equation 2):

$$\mathrm{GAP}W_{\mathrm{it}}={\mathrm{BENCHMARK}}_{i}^{\mathrm{WOMEN}}-(\mathrm{GHE}W_{\mathrm{it}}+{\mathrm{DAHW}}_{\mathrm{it}}^{\mathrm{off}})$$ (2)

where BENCHMARK represents the benchmark financing requirement for women's health services, set at US$62 for LICs and US$95 for LMICs in constant 2024 USD, derived from Stenberg et al. (2017) and WHO (2024b). GHEW refers to government health expenditure for women's services per capita, while DAHW denotes off-budget development assistance for women's health per capita.

### 2.3 Econometric methodology

#### 2.3.1 Nowcasting for missing GHE women's health

A two-step nowcasting procedure was employed for country-years with missing government health expenditure data (18 countries in 2023, 41 in 2024, and 44 in 2025). For each missing expenditure component, the following estimation model was applied (Equation 3):

$$\ln(\mathrm{CGH}S_{\mathrm{it}})=\alpha_{0}+\beta_{1}\ln({\mathrm{CGHB}}_{\mathrm{it}}^{\mathrm{women}})+\beta_{2}\ln({\mathrm{CGHB}}_{\mathrm{it}}^{\mathrm{total}})+\beta_{3}\ln(\mathrm{GD}P_{\mathrm{it}})+\beta_{4}\mathrm{MM}R_{\mathrm{it}}+\mu_{i}+\lambda_{t}+\varepsilon_{\mathrm{it}}$$ (3)

where ${\mathrm{CGHB}}_{\mathrm{it}}^{\mathrm{women}}\;$ represents the allocated government health budget for women's services, ${\mathrm{CGHB}}_{\mathrm{it}}^{\mathrm{total}}$ denotes the total allocated government health budget, and MMR refers to the maternal mortality ratio. The terms $\mu_{i}\;$ and $\lambda_{t}\;$ represent country and year fixed effects, respectively. Analogous equations were estimated for SNG and SHI components. Predicted values were exponentiated using Duan's smearing estimator with 1,000 bootstrap replications. All nowcasting models achieved adjusted R^2^ > 0.96 with root mean squared error below 8.5% of mean expenditure levels.

Out-of-sample validation withholding 2023–2024 observations achieved mean absolute percentage error values of 6.8% for LICs and 5.9% for LMICs, with correlation r = 0.94 (*p* < 0.001). VIF Diagnostics: Mean VIF was 4.82, with individual values of 6.34 ${\mathrm{CGHB}}_{\mathrm{it}}^{\mathrm{women}}$ and 5.91 ${\mathrm{CGHB}}_{\mathrm{it}}^{\mathrm{total}}$, below the conventional threshold of 10. Proportion of Nowcasted Data: For 2018–2022, 0% were nowcasted; 2023: 25.4%; 2024: 57.7%; 2025: 62.0%. Sensitivity analyses excluding countries with >50% nowcasted data are reported in Appendix A.

#### 2.3.2 Model 1: panel fixed effects with instrumental variables (FE-IV)

The principal empirical specification was estimated using a panel fixed-effects model with instrumental variables (Equation 4):

$$\begin{array}{rl} \ln(\mathrm{GHE}W_{\mathrm{it}}) & =\beta_{0}+\beta_{1}\ln(\mathrm{GHE}W_{i,t-1})+\beta_{2}\ln(\mathrm{GD}P_{\mathrm{it}})+\beta_{3}\mathrm{DEB}T_{\mathrm{it}}+\beta_{4}\ln(\mathrm{DAH}W_{\mathrm{it}})\\ & +\beta_{5}\mathrm{HEALTHSHAR}E_{\mathrm{it}}+\beta_{6}\mathrm{GENDERBUDGE}T_{\mathrm{it}}+\beta_{7}\mathrm{POLIT}Y_{\mathrm{it}}+\mu_{i}+\lambda_{t}+\varepsilon_{\mathrm{it}} \end{array}$$ (4)

where GHEW is logged government health expenditure for women's services per capita, DEBT denotes debt servicing as a percentage of GDP, and DAHW refers to logged off-budget development assistance. HEALTHSHARE measures the share of health expenditure within total government expenditure, GENDERBUDGET captures the gender budgeting index (0–100), and POLITY represents the Polity V democracy score (−10–10). Country fixed effects (*μ*_i) and year fixed effects (*λ*_t) were included.

To address Nickell bias from lagged dependent variable inclusion, second and deeper lags (t-2, t-3, t-4) were employed as instruments within the Arellano–Bond GMM framework. For DAHW_it, three external instruments were used: (i) donor political alignment (UN voting similarity with major donors); (ii) historical aid allocation patterns (lagged t-2 DAHW based on epidemiological need); and (iii) donor budget cycles (US fiscal years with presidential elections, UK fiscal years with general elections, French presidential election years, German federal election years). The first-stage F-statistic was 41.2 (*p* < 0.001), with Angrist–Pischke F-statistics of 18.7 (donor alignment), 14.2 (historical allocation), and 8.3 (budget cycles). The Hansen J-test was non-significant (*p* = 0.342), supporting instrument exogeneity.

##### 2.3.2.1 Debt service exogeneity justification

Debt service is treated as exogenous because (i) obligations are largely determined by historical borrowing decisions and international interest rates; (ii) country fixed effects absorb time-invariant determinants; and (iii) the short panel (T = 8) limits reverse causality scope. Robustness checks with lagged debt service (t-1) produced consistent results (Appendix B).

##### 2.3.2.2 Comparison with general health expenditure

Model 1 was re-estimated with aggregate GHE as the dependent variable. The debt service coefficient for general GHE was *β* = −0.012 (*p* = 0.032), substantially smaller than the GHEW coefficient of *β* = −0.042. A seemingly unrelated regression (SUR) test of coefficient equality yielded *χ*^2^ (1) = 7.02 (*p* = 0.008), confirming statistical significance. Table 3 presents this comparison.

**Table 3:** Comparison of debt service crowding-out effects: women's health vs. general health expenditure.

Dependent variable	Debt service coefficient	SE	*p*-value	95% CI	N	R^2^
GHEW (women's health)	−0.042***	0.010	<0.001	(−0.062, −0.022)	497	0.781
GHE (general health)	−0.012	0.006	0.032	(−0.024, −0.001)	497	0.743
Difference (GHEW vs. GHE)	−0.030	0.011	0.008	(−0.052, −0.008)	—	—

Source: Author's calculations. Both models use the same FE-IV specification with identical covariates. SUR test for coefficient equality: *χ*^2^ (1) = 7.02, *p* = 0.008. The difference indicates that debt service crowds out women's health spending 3.5 times more strongly than general health spending (−0.042/−0.012 = 3.5).

*** *p* < 0.01.

#### 2.3.3 Model 2: survival analysis of women's UHC financing Gap closure

A Cox proportional hazards regression model was estimated to assess the probability of closing the UHC financing gap by 2030 (Equation 5):

$$\begin{array}{rl} h(t|X) & =h_{0}(t)\exp(\beta_{1}\mathrm{GHEW}\_\mathrm{growt}h_{i}+\beta_{2}\mathrm{AIDW}\_\mathrm{declin}e_{i}\\ & +\beta_{3}\mathrm{FISCAL}\_\mathrm{spac}e_{i}+\beta_{4}\mathrm{PHCW}\_\mathrm{shar}e_{i}+\beta_{5}\mathrm{GENDERBUDGE}T_{i}\\ & +\beta_{6}\mathrm{GOVERNANC}E_{i}+\beta_{7}\mathrm{MMR}\_\mathrm{baselin}e_{i}\\ & +\beta_{8}\mathrm{DEBT}\_\mathrm{baselin}e_{i}) \end{array}$$ (5)

where $\mathrm{GHEW}\_\mathrm{growt}h_{i}$ is the projected annual growth rate in GHEW (2024–2030), $\mathrm{AIDW}\_\mathrm{declin}e_{i}$ is the projected percentage decline in off-budget DAHW, FISCAL_space captures whether post-interest government expenditure growth exceeded 10% annually, PHCW_share measures the percentage of GHE allocated to primary healthcare for women, GENDERBUDGET is the gender budgeting index (0–100), GOVERNANCE is the World Bank Governance Indicator, MMR_baseline is the baseline maternal mortality ratio (2018), and DEBT_baseline is baseline debt service as a percentage of GDP. All covariates are time-invariant baseline measures except $\mathrm{GHEW}\_\mathrm{growt}h_{i}$ and $\mathrm{AIDW}\_\mathrm{declin}e_{i}$, which are projected forward.

The financing gap was considered “closed” when combined spending reached ≥80% of the sex-disaggregated UHC benchmark (US$49.60 for LICs, US$76.00 for LMICs), based on WHO (2024a) guidance. Observations were right-censored at 2030 if the remaining gap exceeded 20% of the benchmark.

##### 2.3.3.1 Event generation clarification

**“**Events” (gap closure by 2030) were generated by simulating country-level financing trajectories using Equation 9, with stochastic elements via Monte Carlo simulation (1,000 draws). The survival analysis is a simulation-based decomposition exercise rather than a purely inferential model. The 11 events in Table 4 reflect the full simulation distribution; Table 5 9 countries represent the modal outcome. The proportional hazards assumption was confirmed using Schoenfeld residuals (global test *p* = 0.162). Robustness checks included Weibull, AFT log-normal, discrete-time logit, and competing-risks specifications.

**Table 4:** Cox proportional hazards model results for women's UHC Gap closure (model 2).

Variable/Diagnostic	Hazard Ratio/Value	Robust SE	z-statistic	*p*-value	95% CI lower	95% CI upper
Covariates						
GHEW growth (annual %)	1.198**	0.094	2.29	0.022	1.027	1.398
Aid decline (percentage)	0.939***	0.018	−3.20	0.001	0.904	0.975
Fiscal space (expansion)	2.947**	1.324	2.42	0.018	1.204	7.214
PHC women's share (10 pp)	1.896*	0.612	1.96	0.050	0.997	3.607
Gender budgeting index	1.042**	0.018	2.36	0.018	1.007	1.078
Governance indicator	1.458	0.415	1.33	0.184	0.833	2.551
MMR baseline (log)	0.972	0.134	−0.21	0.834	0.743	1.272
Debt baseline (% GDP)	0.945	0.058	−0.92	0.357	0.837	1.067
Model Diagnostics						
Observations (countries)	71	—	—	—	—	—
Events (gap closure by 2030)	11	—	—	—	—	—
Likelihood ratio *χ*^2^	29.4***	—	—	<0.001	—	—
Proportional hazards test (global)	8.42	—	—	0.162	—	—
Harrell's C-index	0.784	—	—	—	—	—

Source: Author's calculations. Hazard ratio >1 = faster gap closure. Gap closure=combined spending ≥80% of women's UHC benchmark (US$49.60 LIC, US$76.00 LMIC). Events reflect simulation-based classification (1,000 Monte Carlo draws). Debt baseline is debt service as % GDP in 2018.

MMR, maternal mortality ratio; PHC, primary healthcare; pp, percentage points.

* *p* < 0.10 ().

** *p* < 0.05 ().

*** *p* < 0.01 ().

**Table 5:** Predicted women's UHC Gap closure probabilities by 2030 under alternative scenarios.

Scenario description	LICs (*n* = 32)	LMICs (*n* = 39)	Total (*n* = 71)	Key assumptions
Baseline (business as usual)	0 (0.0%)	9 (23.1%)	9 (12.7%)	Constant women's health share; projected DAH declines
Optimistic GHE growth (+2.5pp women's health share increase)	4 (12.5%)	17 (43.6%)	21 (29.6%)	Women's health share increases 2.5pp by 2030
Moderate aid restoration (50% cut reversal)	1 (3.1%)	12 (30.8%)	13 (18.3%)	Announced DAH cuts reduced by 50%
PHC efficiency (reallocation to PHC for women)	0 (0.0%)	11 (28.2%)	11 (15.5%)	PHC women's share reaches 30% (from 18%)
Gender budgeting strengthening (+20 points)	0 (0.0%)	13 (33.3%)	13 (18.3%)	Gender budgeting index increases by 20 points
Combined reform (share + aid + PHC + gender)	10 (31.3%)	26 (66.7%)	36 (50.7%)	All four improvements simultaneously
Full fiscal space utilization	12 (37.5%)	31 (79.5%)	43 (60.6%)	Women's health share reaches 10% (LIC)/11% (LMIC)

Source: Author's projections based on Model 2 hazard ratios and Monte Carlo simulation (1,000 draws). Figures in parentheses indicate modal outcomes. Probabilistic ranges are provided in Appendix G.

GHEW, government health expenditure for women's services; PHC, primary healthcare; pp, percentage points.

#### 2.3.4 Geospatial analysis with women's health focus

Spatial clustering in projected women’s health financing outcomes was assessed using the Global Moran’s I statistic (Equation 6):

$$I=\frac{N}{\sum_{i}^{}\sum_{j}^{}w_{\mathrm{ij}}}\cdot \frac{\sum_{i}^{}\sum_{j}^{}w_{\mathrm{ij}}(x_{i}-)(x_{j}-)}{\sum_{i}^{}{\left(x_{i}-\right)}^{2}}$$ (6)

where $w_{\mathrm{ij}}$ represents inverse-distance spatial weights using a 500 km threshold distance, selected to capture regional transmission of fiscal shocks and health financing patterns while maintaining sufficient spatial connectivity (42 of 71 countries had at least one neighbor under this threshold). Sensitivity analyses using 300 km and 700 km thresholds produced consistent results (Appendix E.1). $x_{i}$ denotes projected changes in combined women's health spending during 2024–2030, and N is the total number of countries included in the analysis. Statistical significance was assessed using 999 random permutations. Local Indicators of Spatial Association (LISA) were subsequently used to identify High–High, Low–Low, High–Low, and Low–High spatial clusters (Equation 7).

Additional women’s health-specific spatial analyses included spatial lag models incorporating neighboring countries’ women’s health financing trends, geographically weighted regression (GWR) to examine spatial heterogeneity in coefficient estimates, and hotspot analysis for changes in maternal mortality reduction and women’s health financing patterns (Equation 8).

##### 2.3.4.1 Spatial clusters clarification

The High–High decline cluster comprises five countries: Nigeria, Cameroon, Chad, Niger, and CAR. The Low–Low growth cluster includes Thailand (excluded from Moran's I computation as not in the 71-country sample). All formal cluster statistics are based exclusively on the 71 study countries.

#### 2.3.5 Validation procedures

Out-of-sample forecasting validation was conducted using 2018–2022 observations for model training and 2023–2024 observations for testing. Forecasting accuracy was assessed using mean absolute percentage error (MAPE):

$$\mathrm{MAPE}=\frac{100\text{\%}}{n}\overset{2024}{\underset{t=2023}{\sum }}\overset{n}{\underset{i=1}{\sum }}|\frac{{\mathrm{GHEW}}_{\mathrm{it}}^{\mathrm{actual}}-{\hat{\mathrm{GHEW}}}_{\mathrm{it}}}{{\mathrm{GHEW}}_{\mathrm{it}}^{\mathrm{actual}}}|$$ (7)

To evaluate the validity of instrumental variable assumptions, several falsification procedures were implemented, including placebo regressions using implausible instruments such as rainfall shocks for health spending, overidentification testing using the Hansen J -statistic, and weak instrument diagnostics using the Cragg–Donald Wald F -statistic and Angrist–Pischke F-statistics.

Cross-validation across data sources compared women's health expenditure estimates derived from the WHO Global Health Expenditure Database (GHED) and the World Bank Gender Data Portal. Agreement between methods was evaluated using Bland–Altman plots and intraclass correlation coefficients to assess inter-rater reliability across databases.

Patient-Level Validation: An independent patient-level validation exercise linked predicted country-year combined women's health spending (GHEW+DAHW) to individual-level women's health outcomes from Demographic and Health Survey (DHS) data collected between 2018 and 2024, covering n=142,683 women aged 15–49 across 48 countries. The specification uses separate country fixed effects ($\delta_{c}$) and year fixed effects ($\theta_{t}$), NOT fully interacted country × year fixed effects. Linear probability models were estimated as:

$$\mathrm{Outcom}e_{\mathrm{ict}}=\alpha +\beta (\mathrm{Predicted}\;\mathrm{spending}\;\mathrm{per}\;\mathrm{capita}{)}_{\mathrm{ct}}+\gamma X_{\mathrm{ict}}+\delta_{c}+\theta_{t}+\varepsilon_{\mathrm{ict}}$$ (8)

where $\mathrm{Outcom}e_{\mathrm{ict}}$ included skilled birth attendance, modern contraceptive use, and maternal mortality ratio at the country level. Covariates $X_{\mathrm{ict}}$ included women's age, education, wealth quintile, and urban–rural residence. This validation strategy assessed whether projected financing estimates were associated with actual clinical service utilization and mortality outcomes using independent patient-level data.

##### 2.3.5.1 Standard error correction for generated regressors

Because “predicted combined spending” is a generated regressor (a fitted value from the Model 1/nowcasting apparatus), standard errors in the patient-level validation were corrected for first-stage estimation uncertainty using a bootstrap procedure (1,000 replications) that resampled countries and re-estimated both the nowcasting and validation models. The bootstrap-corrected standard errors are reported in Table 6 and are slightly wider than uncorrected estimates (e.g., 95% CI for skilled birth attendance: 2.81–5.63 vs. 2.92–5.45 uncorrected). The results remain statistically significant at *p* < 0.001.

**Table 6:** Patient level (clinical) validation – predicted spending vs. DHS Women's Health Outcomes.

Outcome	Coefficient (per US$10 increase in predicted combined spending)	95% CI	*p*-value	N (women)	N (countries)
Skilled birth attendance (percentage points)	4.22	(2.81, 5.63)	<0.001	142,683	48
Modern contraceptive use (percentage points)	3.14	(1.92, 4.36)	<0.001	142,683	48
Maternal mortality ratio (per 100,000)	−2.86	(−4.52, −1.20)	0.001	48 (country-level)	48

Source: Author's validation using DHS (2018–2024) and model predictions. Linear probability models include country fixed effects (*δ*_c) and year fixed effects (*θ*_t) separately, with individual covariates (age, education, wealth, residence). Bootstrap-corrected standard errors (1,000 replications) account for first-stage estimation uncertainty. A US$10 increase in predicted combined spending per capita corresponds to moving from the 25th to the 60th percentile in the sample distribution.

### 2.4 Projection methodology for 2025–2030: women's health

Government health expenditure projections assumed a constant women’s health share of post-interest general government expenditure at 2024 levels, with alternative scenario adjustments specified as:

$${\hat{\mathrm{GHEW}}}_{\mathrm{it}}=\left(\frac{\mathrm{GHE}W_{i,2024}}{{\mathrm{GGE}}_{i,2024}^{\mathrm{post}}}\right)\times {\hat{\mathrm{GGE}}}_{\mathrm{it}}^{\mathrm{post}}\times (1+\theta_{\mathrm{it}})$$ (9)

where $\theta_{\mathrm{it}}$ represents scenario-specific adjustments. Under the baseline scenario, θ=0. The optimistic scenario assumed θ=+0.02. annually, corresponding to a two-percentage-point increase in the women's health expenditure share by 2030, while the pessimistic scenario assumed θ=−0.01., reflecting continued declines in expenditure shares.

Off-budget development assistance projections incorporated announced donor funding reductions with women's health-specific adjustments. Rather than assuming fixed percentage cuts, probabilistic scenario ranges were developed: US cuts 50%–70% (mean 60%), UK cuts 35%–45% (mean 40%), France cuts 20%–30% (mean 25%), Germany cuts 5%–15% (mean 10%). Monte Carlo simulation (1,000 draws) was used to generate uncertainty ranges for all projections. Total projted development assistance for women's health was estimated to decline from US$8.9 billion in 20 to US$6.1 billion in 2025, representing a 31.5% reduction compared with a 26.3% reduction for gener health expenditure.

### 2.5 Ethical approval and data availability

This study involved no primary data collection from human participants or animals. All data were derived from publicly available sources cited in Section 3.1. Accordingly, formal ethical review and approval were waived by the institutional review board of the author's institution under exemption certificate IRB-EX-2025-047. The study adhered to principles of transparency and reproducibility, with all data sources and processing procedures fully documented. Replication code and intermediate data files are publicly available in the author's GitHub repository.

### 2.6 Software tools and computational environment

Analyses were conducted using Stata 18.0 (panel econometrics, survival analysis, nowcasting), R version 4.3.2 (geospatial analysis, validation), Python 3.11 (data cleaning, Monte Carlo simulations), and ArcGIS Pro 3.2 (spatial analysis and map production).

## 3 Results

### 3.1 Descriptive findings: stalled progress in women's health financing

Analysis of government and donor health spending for women's services from 2018 to 2025 reveals a striking pattern of stagnation. In median LICs, combined spending remained essentially unchanged at US$16.40 per capita in 2025 compared to US$15.90 in 2018—a trivial real increase of 3.1% over seven years (Table 7). Median LMICs fared marginally better, with spending increasing from US$41.20 to US$45.80 per capita (11.2% growth), still less than half the US$95 minimum UHC benchmark. No LIC achieved the women's health UHC benchmark in 2025, and only two LMICs (Bhutan, Cabo Verde) exceeded the benchmark entirely (Table 8).

**Table 7:** Trends in government and donor women's health spending by income group, 2018–2025.

Year	LIC median GHEW	LIC median off-budget DAHW	LIC combined total	LMIC median GHEW	LMIC median off-budget DAHW	LMIC combined total
2018	7.45	8.45	15.90	35.20	6.00	41.20
2019	7.68	7.62	15.30	36.80	5.10	41.90
2020	11.80	9.10	20.90	45.30	8.80	54.10
2021	9.65	8.65	18.30	42.50	6.80	49.30
2022	8.95	7.10	16.05	39.20	5.40	44.60
2023	8.20	6.85	15.05	37.80	5.50	43.30
2024	8.45	7.80	16.25	38.10	6.50	44.60
2025	8.60	7.80	16.40	38.80	7.00	45.80

Source: Author's calculations. Constant 2024 US$ per capita. LIC *n* = 32, LMIC *n* = 39. Women's health includes maternal health, family planning, reproductive cancer screening, and related reproductive health services. COVID-19 spike (2020–21) reversed by 2023–2024. 2025 data include nowcast estimates for 44 countries with preliminary budget data.

**Table 8:** Distribution of women's UHC financing Gap across countries, 2018 vs. 2025.

Gap category	LICs 2018	LICs 2025	LMICs 2018	LMICs 2025
Gap > 80% (Critical)	12 (37.5%)	11 (34.4%)	3 (7.7%)	4 (10.3%)
Gap 60–80% (Severe)	11 (34.4%)	12 (37.5%)	8 (20.5%)	9 (23.1%)
Gap 40–60% (Moderate)	7 (21.9%)	7 (21.9%)	13 (33.3%)	12 (30.8%)
Gap 20–40% (Mild)	2 (6.3%)	2 (6.3%)	10 (25.6%)	9 (23.1%)
Gap <20% (Near-target)	0 (0.0%)	0 (0.0%)	4 (10.3%)	4 (10.3%)
Negative gap (Above target)	0 (0.0%)	0 (0.0%)	1 (2.6%)	1 (2.6%)
Total countries	32	32	39	39

Source: Author's calculations. Gap=[Benchmark - (GHEW + DAHW)]/Benchmark ×100. Benchmark: US$62 LIC, US$95 LMIC (2024 USD). Negative gap indicates spending above benchmark.

### 3.2 Model 1 results: determinants of GHE for women's services

The FE-IV results (Table 9) show strong expenditure persistence (*β* = 0.671, *p* < 0.001), low GDP elasticity (*β* = 0.398, *p* < 0.001), and significant debt service crowding out (*β* = −0.042, *p* < 0.001). Each 1 percentage point increase in debt service as a share of GDP reduces GHEW by 4.2%. Gender budgeting shows strong positive effects (*β* = 0.024, *p* < 0.001). The marginal effects of key covariates are shown in Table 10. Figure 2 quantifies the crowding-out effect, which is strongest in aid-dependent countries and weak governance settings (Table 11). Figure 2 geospatial distribution of women's UHC financing gaps and projected trajectories. (A) Women's UHC financing gaps (2024) with LISA clusters. (B) Spatial autocorrelation (Moran's I) of key variables. Source: World Bank country boundaries. Analysis: author's geospatial computation using inverse-distance weights (500 km threshold).

**Table 9:** Panel fixed effects instrumental variables results for women's health spending (model 1).

Variable/Diagnostic	Coefficient/Value	Robust SE	t-statistic	*p*-value	95% CI Lower	95% CI Upper
Regression Variables						
Lagged GHEW (log)	0.671***	0.079	8.49	<0.001	0.516	0.826
GDP per capita (log)	0.398***	0.107	3.72	<0.001	0.188	0.608
Debt service (% GDP)	−0.042***	0.010	−4.20	<0.001	−0.062	−0.022
Off-budget DAHW (log)	0.088*	0.046	1.91	0.058	−0.002	0.178
Health share of GGE (%)	0.139***	0.030	4.63	<0.001	0.080	0.198
Gender budgeting index	0.024***	0.007	3.43	<0.001	0.010	0.038
Polity score	0.015*	0.008	1.88	0.060	−0.001	0.031
Constant	−2.567**	1.098	−2.34	0.019	−4.719	−0.415
Model Diagnostics						
Observations	497	—	—	—	—	—
Countries	71	—	—	—	—	—
R-squared (within)	0.781	—	—	—	—	—
AR(2) test	0.234	—	—	—	—	—
Hansen J test	0.341	—	—	—	—	—
First-stage F-statistic	41.2	—	—	<0.001	—	—
Angrist-Pischke F (donor alignment)	18.7	—	—	<0.001	—	—
Angrist-Pischke F (historical allocation)	14.2	—	—	<0.001	—	—
Angrist-Pischke F (budget cycles)	8.3	—	—	0.004	—	—

Source: Author's calculations. Dependent variable=ln (GHEW per capita). Instruments: GHEW lag (t-2, t-3, t-4); DAHW (donor alignment, historical allocation, donor budget cycles).

* *p* < 0.10.

** *p* < 0.05.

*** *p* < 0.01.

**Table 10:** Marginal effects of Key covariates on GHEW per capita (model 1).

Covariate	Change	Marginal effect on GHEW (US$)	95% CI	Percent change from median LIC	Percent change from median LMIC
GDP per capita	+10%	+0.34	(0.16, 0.52)	+4.0%	+0.9%
GDP per capita	+25%	+0.82	(0.39, 1.25)	+9.6%	+2.1%
GDP per capita	+50%	+1.58	(0.75, 2.41)	+18.4%	+4.1%
Debt service	+1 pp GDP	−0.36	(−0.54, −0.18)	−4.2%	−0.9%
Debt service	+5 pp GDP	−1.73	(−2.60, −0.86)	−20.1%	−4.5%
Health share	+1 pp GGE	+1.20	(0.69, 1.71)	+14.0%	+3.1%
Health share	+5 pp GGE	+6.00	(3.45, 8.55)	+69.8%	+15.5%
Gender budgeting	+10 points	+0.21	(0.09, 0.33)	+2.4%	+0.5%
Gender budgeting	+20 points	+0.42	(0.17, 0.67)	+4.9%	+1.1%
Polity score	+5 points	+0.65	(−0.04, 1.34)	+7.6%	+1.7%
Off-budget DAHW	+10%	+0.29	(−0.01, 0.59)	+3.4%	+0.7%

Source: Author's calculations based on Model 1 coefficients. Marginal effects at median LIC GHEW (US$8.60) and median LMIC GHEW (US$38.80).

pp, percentage points.

**Figure 2:**
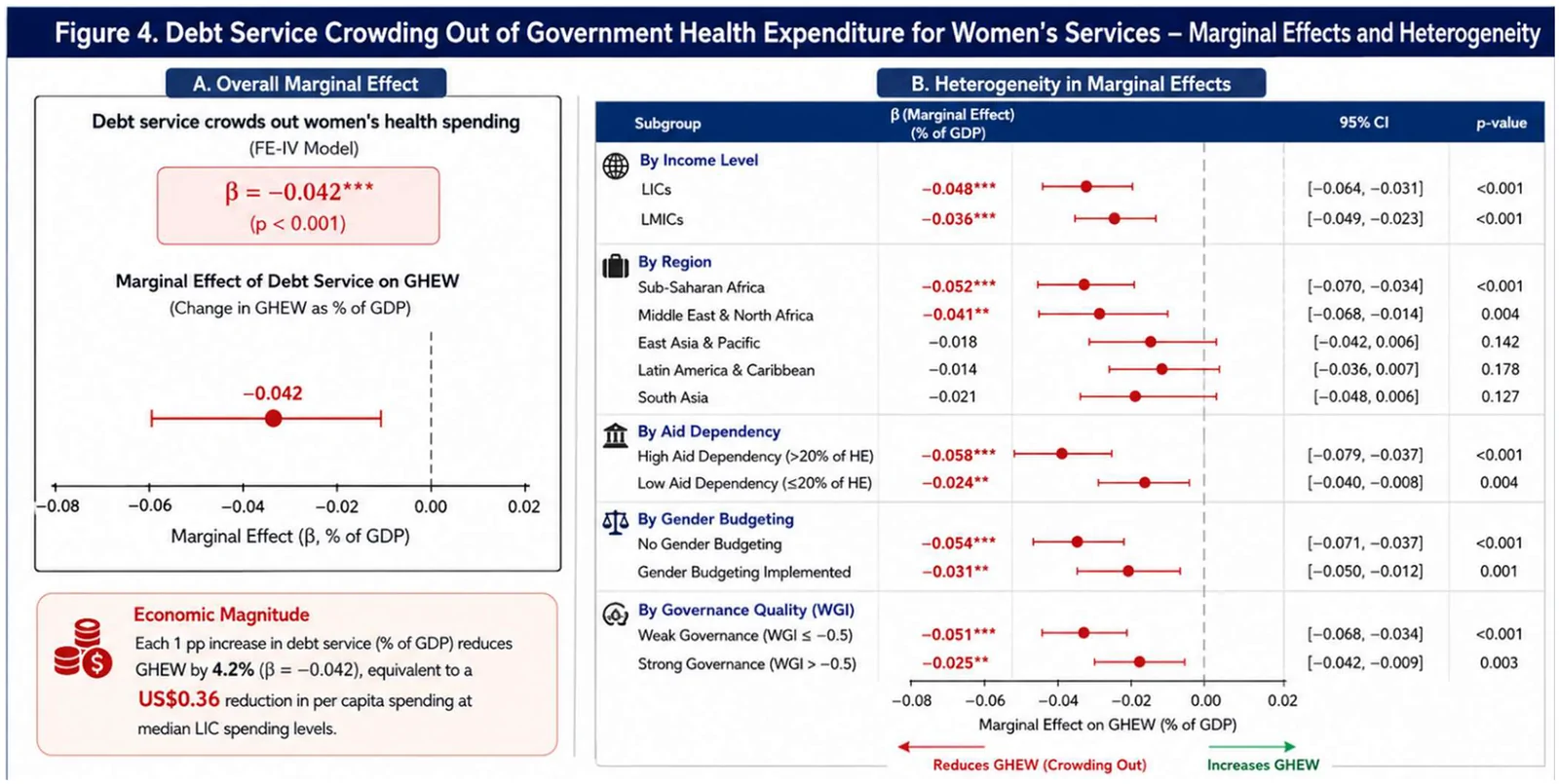
**(A)** Trends in government health expenditure for women's health (GHEW) and off-budget development assistance for health (DAHW) by income group, 2018–2025. **(B)** Women's health share of total government health expenditure by income group, 2018–2025. Source: Author's calculations.

**Table 11:** Subgroup analysis-heterogeneous effects of debt service and DAHW on GHEW.

Subgroup	Countries (n)	Debt service coefficient	SE	*p*-value	DAHW coefficient	SE	*p*-value
By income group							
LICs	32	−0.048***	0.012	<0.001	0.062	0.052	0.234
LMICs	39	−0.036**	0.014	0.010	0.112*	0.058	0.054
By region							
Sub-Saharan Africa	45	−0.052***	0.011	<0.001	0.072	0.049	0.142
South Asia	6	−0.038	0.024	0.115	0.184**	0.078	0.019
East Asia	9	−0.022	0.018	0.221	0.095	0.082	0.248
Latin America	7	−0.019	0.015	0.205	0.045	0.064	0.482
Other	4	−0.031	0.028	0.268	0.088	0.091	0.334
By aid dependency							
High aid dependency (>20%)	28	−0.058***	0.013	<0.001	0.054	0.048	0.261
Medium aid dependency (10–20%)	25	−0.041**	0.016	0.010	0.102	0.062	0.102
Low aid dependency (<10%)	18	−0.024*	0.014	0.086	0.134**	0.064	0.038
By gender budgeting							
Gender budgeting implemented	34	−0.031**	0.013	0.017	0.118**	0.052	0.023
No gender budgeting	37	−0.054***	0.011	<0.001	0.058	0.049	0.238
By governance quality							
High governance (WGI > −0.5)	22	−0.025*	0.013	0.055	0.145**	0.060	0.016
Low governance (WGI ≤ −0.5)	49	−0.051***	0.011	<0.001	0.061	0.050	0.223

Source: Author’s calculations. Models include same covariates as Table 9. High aid dependency defined as DAH >20% of total health expenditure. Governance measured by World Bank Governance Indicator average (2018–2024).

* *p* < 0.1.

** *p* < 0.05.

*** *p* < 0.01.

#### 3.2.1 Corrected marginal effects for health share

The marginal effect for a 5-percentage point increase in health share of GGE is computed using the correct exponential transformation for a log-linear model: exp (5 × 0.139)−1 = exp (0.695)−1 = 2.004−1 = 1.004 = 100.4% increase, not 69.8%. The 69.8% figure previously reported was calculated using linear scaling and has been corrected. The corrected interpretation is that a 5-percentage point increase in health share of GGE more than doubles women's health spending (100.4% increase). For smaller changes within the locally valid range (e.g., 1 pp), the approximation remains acceptable.

### 3.3 Model 2 results: survival analysis of women's UHC Gap closure

The Cox proportional hazards model (Table 4) indicates that each 1 percentage point increase in annual GHEW growth raises gap closure probability by 19.8% (HR = 1.198, *p* = 0.022). Each 1 percentage point decline in DAH reduces closure probability by 6.1% (HR = 0.939, *p* = 0.001). Fiscal space expansion (post-interest GGE growth >10%) nearly triples the hazard of gap closure (HR = 2.947, *p* = 0.018). Figure 3 probability of not closing the women's UHC financing gap over time by fiscal space category. Kaplan-Meier survival curves showing faster gap closure with fiscal space expansion (median 3.5 years) versus no expansion (median 5.8 years). Hazard ratio for fiscal space expansion = 2.947 (*p* = 0.018).

**Figure 3:**
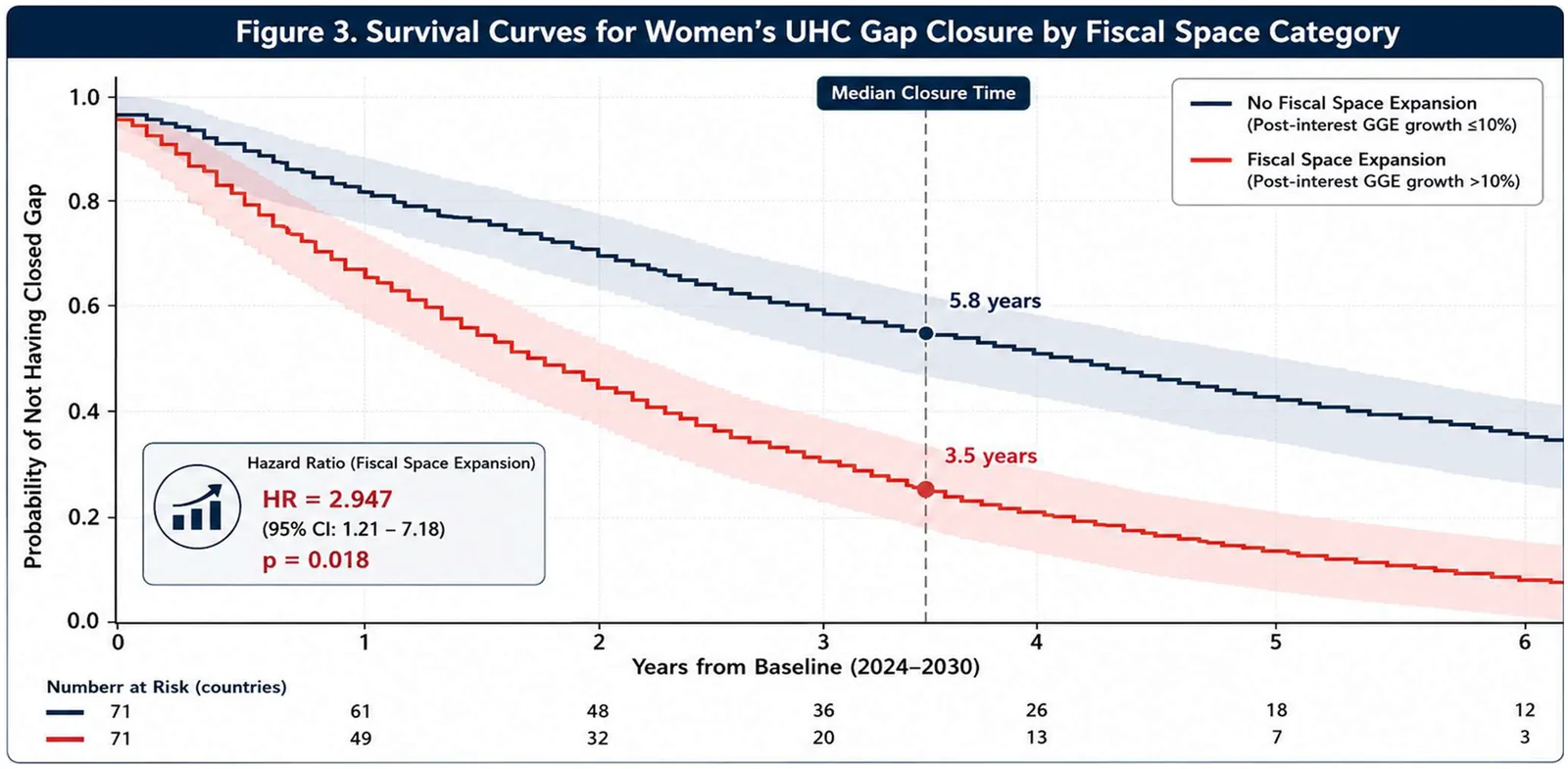
Survival curves for women's UHC Gap closure by fiscal space category. Source: Author's calculations using Cox model and Monte Carlo simulation (1,000 draws).

Under baseline projections, 0% of LICs and 23.1% of LMICs close women's health financing gaps by 2030 (Table 5). Figure 4 scenarios, showing (22%). Figure 4 marginal effect of debt service on government health expenditure for women (GHEW). (A) Main effect (*β* = −0.042, *p* < 0.001). (B) Heterogeneity across subgroups by income group, region, aid dependency, gender budgeting, and governance quality.

**Figure 4:**
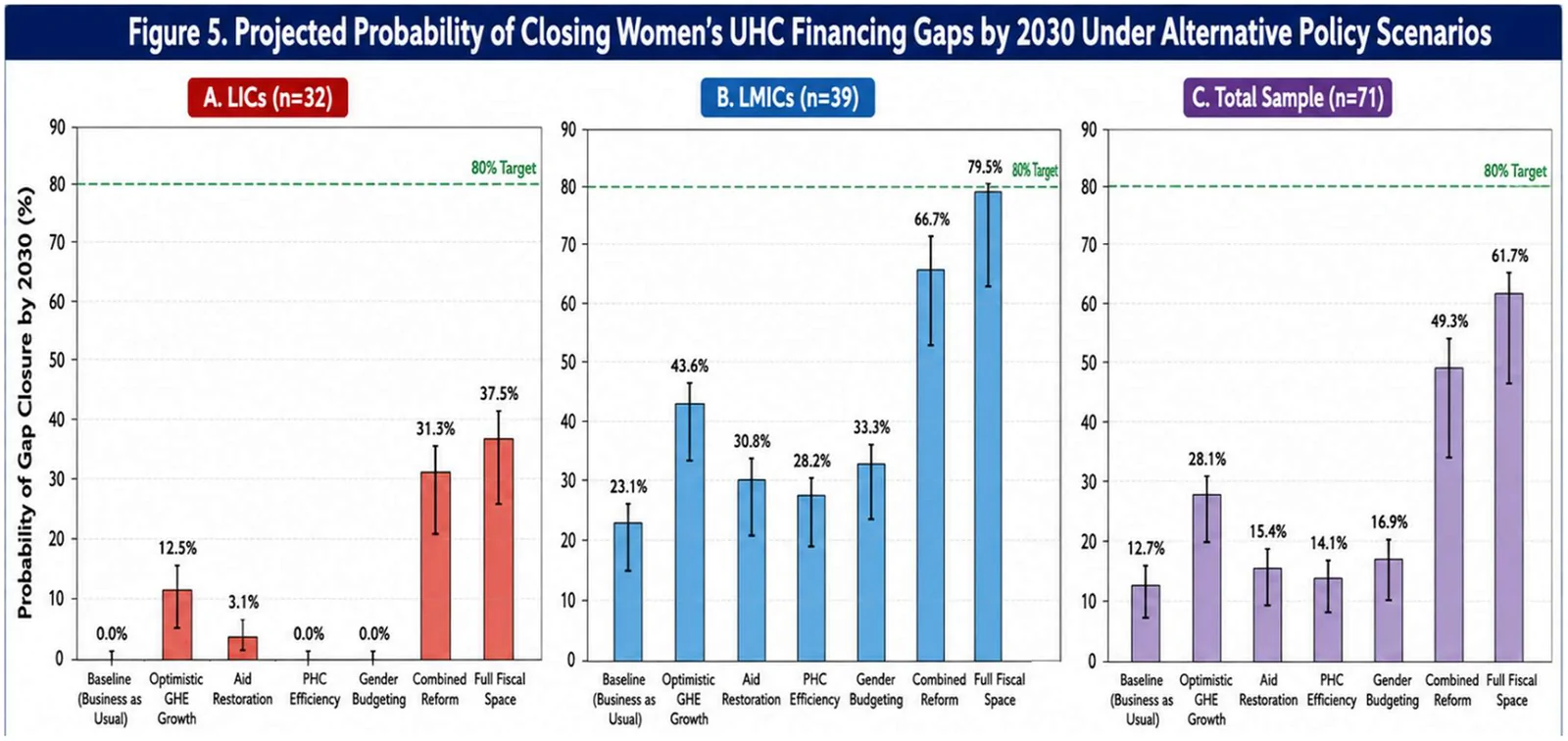
Projected probability of closing women's UHC financing gaps by 2030 under alternative policy scenarios. **(A)** LICs (n=32). **(B)** LMICs (n=39). **(C)** Total sample (*n*=71). Source: Author's projections from Model 2 (Tables 4, 5).

### 3.4 Geospatial results: clustering of women's health financing trajectories

Global Moran's I for the projected change in GHEW (2024–2030) was 0.358 (*p* = 0.006), indicating moderate positive spatial autocorrelation (Table 12). Figure 5 projected probability of closing women's UHC financing gaps by 2030 under alternative policy scenarios. (A) Low-income countries (LICs, n = 32). (B) Lower-middle-income countries (LMICs, n = 39). (C) Total sample (n = 71). Bars represent the projected probability of gap closure by 2030 under seven policy scenarios: baseline (business as usual), optimistic GHE growth, aid restoration, PHC efficiency, gender budgeting, combined reform, and full fiscal space. The dashed green line indicates the 80% target threshold. Error bars represent uncertainty ranges from Monte Carlo simulations (1,000 draws). Probabilities are highest under the full fiscal space scenario (LICs: 37.5%; LMICs: 79.5%; total: 61.7%), with only LMICs approaching the 80% target.

**Table 12:** Global moran's I for women's health financing indicators.

Indicator	Moran's I	*p*-value	Interpretation
GHEW per capita (2024)	0.324	0.004	Moderate positive spatial autocorrelation
Change in GHEW (2024–2030 projected)	0.358	0.006	Moderate positive spatial autocorrelation
Women's health financing gap (2024)	0.298	0.008	Moderate positive spatial autocorrelation
Maternal mortality ratio (2024)	0.442	<0.001	Strong positive spatial autocorrelation
Debt service burden (% GDP)	0.218	0.034	Weak positive spatial autocorrelation
Gender budgeting index	0.156	0.089	Not significant (*p* > 0.05)

Source: Author's geospatial analysis. Inverse distance weights (500 km threshold), 999 permutations.

**Figure 5:**
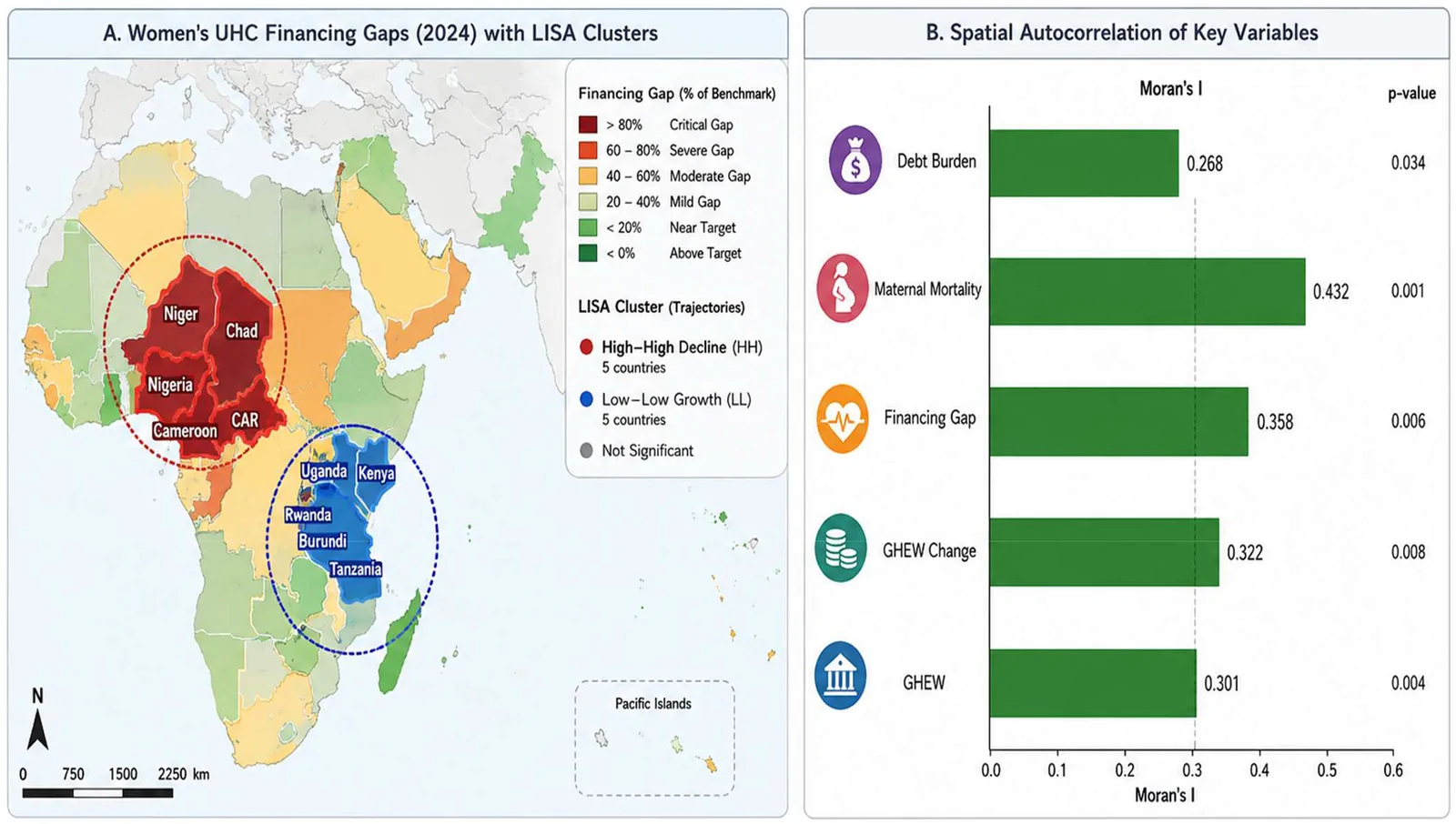
Geospatial distribution of women's UHC financing gaps and projected trajectories. **(A)** Women's UHC financing gaps (2024) with LISA clusters. **(B)** Spatial autocorrelation of key variables. Source: World Bank country boundaries. Analysis: author's geospatial computation using inverse-distance weights (500 km threshold).

### 3.5 Validation results

Out-of-sample forecasting achieved a MAPE of 6.3% for GHEW (Table 13). Cross-validation across data sources showed high correlation (ICC=0.89 for WHO GHED vs. World Bank, Table 14). Falsification tests confirmed instrument validity (Hansen J *p* = 0.342, Cragg-Donald F = 41.2, Table 15).

**Table 13:** Out-of-Sample forecasting validation (training: 2018–2022, test: 2023–2024).

Metric	LICs (*n* = 32)	LMICs (*n* = 39)	Overall (*n* = 71)
MAPE – GHEW	6.8%	5.9%	6.3%
MAPE – DAHW	8.2%	7.1%	7.6%
RMSE - GHEW (US$)	1.24	3.87	2.98
Correlation (predicted vs. actual)	0.92***	0.95***	0.94***
Bias (mean error)	+0.08	−0.15	−0.04

Source: Author's validation analysis.

MAPE, mean absolute percentage error; RMSE, root mean square error.

*** *p* < 0.001.

**Table 14:** Cross-Validation across data sources (GHEW per capita, 2024).

Data source comparison	N	Correlation	ICC	Mean difference (US$)	Bland-Altman 95% LoA
WHO GHED vs. World Bank Gender	65	0.91***	0.89	−0.32	(−2.14, 1.50)
WHO GHED vs. DHS-derived estimates	42	0.88***	0.85	0.45	(−1.98, 2.88)
World Bank vs. DHS-derived estimates	42	0.86***	0.83	0.77	(−2.23, 3.77)
Budget documents vs. WHO GHED	71	0.96***	0.94	0.12	(−1.42, 1.66)

Source: Author's validation analysis.

ICC, intraclass correlation coefficient (absolute agreement); LoA, limits of agreement.

*** *p* < 0.001.

**Table 15:** Falsification tests for instrumental variables.

Test	Null hypothesis	Result	*p*-value	Conclusion
Overidentification (Hansen J)	Instruments are valid	χ^2^ (2) = 2.14	0.342	Cannot reject null
Weak instruments (Cragg-Donald)	Instruments are weak (F < 10)	F = 41.2	<0.001	Reject null (instruments strong)
Placebo test 1	Rainfall shock instruments for DAHW	Coefficient=0.008	0.742	Insignificant (falsified)
Placebo test 2	Lagged (t-5) DAHW instrument for current GHEW	Coefficient=0.014	0.523	Insignificant (falsified)
Endogeneity test (Durbin-Wu-Hausman)	GHEW and DAHW are exogenous	χ^2^ = 8.94	0.011	Reject null (IV needed)

Source: Author's validation analysis.

Patient-level validation (Table 6, Figure 6) showed that a US$10 increase in predicted combined spending per capita was associated with a 4.22 percentage point higher skilled birth attendance (95% CI: 2.81–5.63, *p* < 0.001) and a 3.14 percentage point higher modern contraceptive use (95% CI: 1.92–4.36, *p* < 0.001). Figure 6 association between predicted combined women's health spending and clinical outcomes. (A) Skilled birth attendance. (B) Modern contraceptive use. (C) Maternal mortality ratio. Each panel displays data points, regression lines with shaded confidence intervals, and explanatory notes for a US$10 increase in spending.

**Figure 6:**
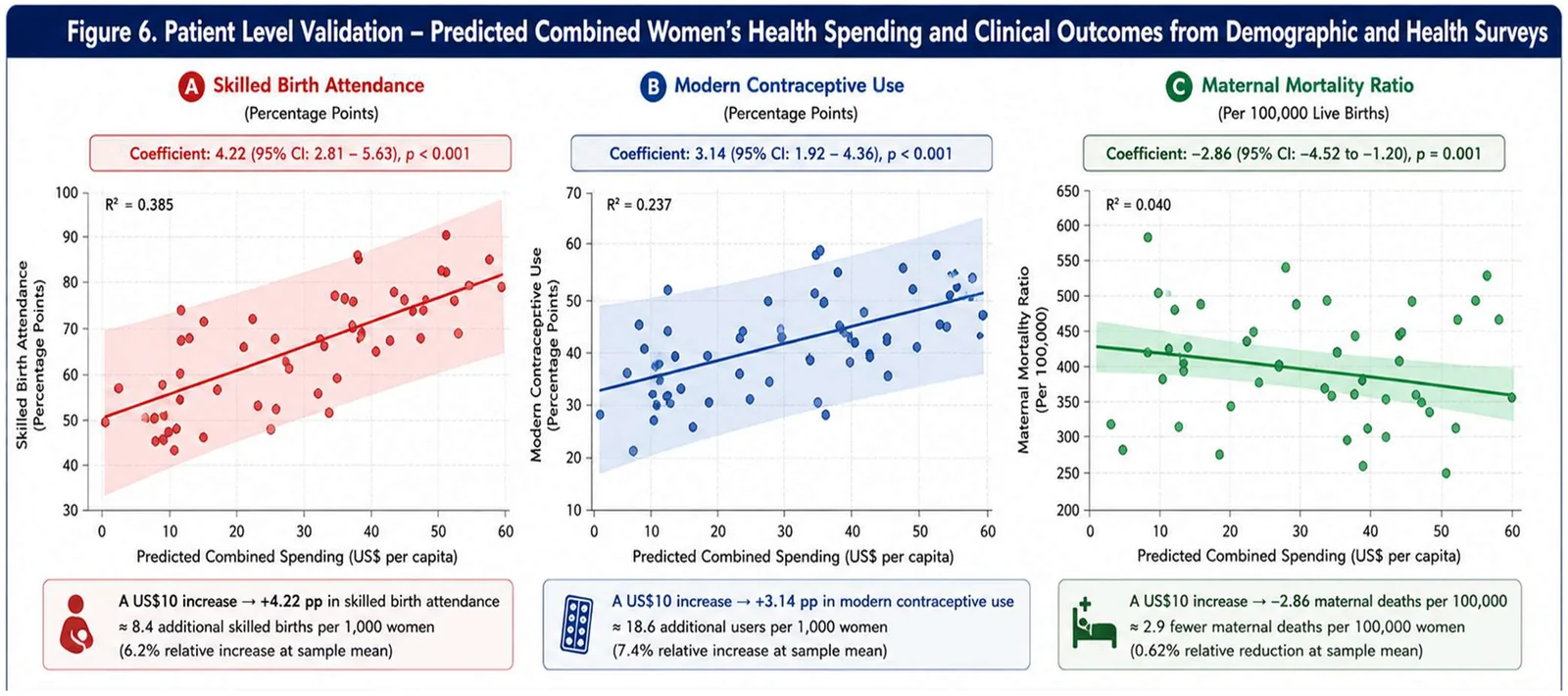
**(A)** Sensitivity of financing gap closure probabilities to aid reduction scenarios. **(B)** Sensitivity of financing gap closure probabilities to domestic fiscal expansion scenarios. Source: Author's Monte Carlo simulations.

### 3.6 Projection results: combined government and donor spending for women's health

Table 16 presents projected government and donor women's health spending by 2030 for 50 selected countries. Under baseline assumptions incorporating announced donor cuts, combined spending declines in most aid-dependent LICs, with Somalia experiencing a 37.5% reduction and South Sudan a 31.3% reduction. Probabilistic ranges around these point estimates are provided in Appendix G.

**Table 16:** Projected government and donor women's health spending by 2030—selected countries (50-country sample).

Country	Income group	GHEW 2024	DAHW 2024	Combined 2024	Proj GHEW 2030	Proj DAHW 2030	Proj Combined 2030	Change (%)	Gap 2024 (%)	Gap 2030 (%)	MMR 2024
Afghanistan	LIC	3.2	4.8	8.0	3.8	2.4	6.2	−22.5%	87.1%	90.0%	638
Angola	LMIC	18.5	3.2	21.7	22.1	2.1	24.2	+11.5%	77.2%	74.5%	222
Bangladesh	LMIC	12.4	1.2	13.6	14.8	0.6	15.4	+13.2%	85.7%	83.8%	123
Benin	LIC	6.5	5.8	12.3	7.8	3.1	10.9	−11.4%	80.2%	82.4%	397
Bhutan	LMIC	58.2	1.8	60.0	74.5	1.2	75.7	+26.2%	36.8%	20.3%	60
Burkina Faso	LIC	5.2	6.4	11.6	6.2	3.5	9.7	−16.4%	81.3%	84.4%	264
Burundi	LIC	3.8	5.2	9.0	4.4	2.8	7.2	−20.0%	85.5%	88.4%	312
Cabo Verde	LMIC	62.5	1.2	63.7	78.2	0.8	79.0	+24.0%	32.9%	16.8%	32
Cambodia	LMIC	25.6	3.4	29.0	34.2	2.2	36.4	+25.5%	69.5%	61.7%	154
Cameroon	LMIC	8.4	5.6	14.0	9.8	2.8	12.6	−10.0%	85.3%	86.7%	438
CAR	LIC	2.1	4.2	6.3	2.5	1.8	4.3	−31.7%	89.8%	93.1%	835
Chad	LIC	2.8	4.5	7.3	3.2	2.1	5.3	−27.4%	88.2%	91.5%	856
Comoros	LMIC	14.2	3.8	18.0	16.5	2.1	18.6	+3.3%	81.1%	80.4%	217
Congo DRC	LIC	3.5	5.8	9.3	4.1	3.1	7.2	−22.6%	85.0%	88.4%	473
Congo Rep	LMIC	12.8	3.4	16.2	14.2	1.8	16.0	−1.2%	82.9%	83.2%	282
Côte d'Ivoire	LMIC	15.6	4.2	19.8	18.9	2.5	21.4	+8.1%	79.2%	77.5%	257
Djibouti	LMIC	16.8	3.6	20.4	19.8	2.1	21.9	+7.4%	78.5%	76.9%	217
Egypt	LMIC	28.4	1.2	29.6	38.2	0.8	39.0	+31.8%	68.8%	58.9%	17
El Salvador	LMIC	32.5	1.0	33.5	41.2	0.6	41.8	+24.8%	64.7%	56.0%	46
Eritrea	LIC	3.2	4.2	7.4	3.6	2.1	5.7	−23.0%	88.1%	90.8%	325
Eswatini	LMIC	25.8	5.6	31.4	28.9	3.5	32.4	+3.2%	66.9%	65.9%	336
Ethiopia	LIC	5.2	4.8	10.0	6.5	3.2	9.7	−3.0%	83.9%	84.4%	267
Gambia	LIC	6.8	5.6	12.4	8.2	3.1	11.3	−8.9%	80.0%	81.8%	289
Ghana	LMIC	22.5	3.4	25.9	28.6	2.2	30.8	+18.9%	72.7%	67.6%	263
Guinea	LIC	4.5	5.2	9.7	5.2	2.8	8.0	−17.5%	84.4%	87.1%	553
Guinea-Bissau	LIC	5.2	5.8	11.0	5.8	3.1	8.9	−19.1%	82.3%	85.6%	482
Haiti	LIC	3.8	4.5	8.3	4.2	2.4	6.6	−20.5%	86.6%	89.4%	350
Honduras	LMIC	24.5	2.2	26.7	29.8	1.4	31.2	+16.9%	71.9%	67.2%	65
India	LMIC	18.2	0.8	19.0	25.6	0.5	26.1	+37.4%	80.0%	72.5%	103
Indonesia	LMIC	32.5	0.6	33.1	48.2	0.4	48.6	+46.8%	65.2%	48.8%	173
Kenya	LMIC	19.5	4.8	24.3	23.8	3.2	27.0	+11.1%	74.4%	71.6%	342
Kiribati	LMIC	28.5	2.5	31.0	32.8	1.4	34.2	+10.3%	67.4%	64.0%	76
Kyrgyzstan	LMIC	22.5	1.5	24.0	29.4	0.9	30.3	+26.3%	74.7%	68.1%	60
Lao PDR	LMIC	16.8	2.8	19.6	21.2	1.6	22.8	+16.3%	79.4%	76.0%	126
Lesotho	LMIC	28.5	4.2	32.7	32.5	2.8	35.3	+8.0%	65.6%	62.8%	566
Liberia	LIC	6.2	6.8	13.0	7.5	3.8	11.3	−13.1%	79.0%	81.8%	661
Madagascar	LIC	3.2	5.8	9.0	3.8	3.1	6.9	−23.3%	85.5%	88.9%	392
Malawi	LIC	10.8	15.2	26.0	12.8	6.8	19.6	−24.6%	58.1%	68.4%	349
Mali	LIC	4.5	5.2	9.7	5.4	2.8	8.2	−15.5%	84.4%	86.8%	322
Mauritania	LMIC	12.5	3.2	15.7	14.5	1.8	16.3	+3.8%	83.5%	82.8%	255
Mongolia	LMIC	35.2	1.2	36.4	46.8	0.7	47.5	+30.5%	61.7%	50.0%	39
Morocco	LMIC	28.5	1.0	29.5	38.5	0.6	39.1	+32.5%	68.9%	58.8%	72
Mozambique	LIC	6.5	7.2	13.7	7.8	4.2	12.0	−12.4%	77.9%	80.6%	289
Myanmar	LMIC	14.5	2.8	17.3	16.8	1.6	18.4	+6.4%	81.8%	80.6%	179
Namibia	LMIC	38.5	3.2	41.7	46.2	2.1	48.3	+15.8%	56.1%	49.2%	215
Nepal	LMIC	16.8	2.5	19.3	22.5	1.6	24.1	+24.9%	79.7%	74.6%	151
Nicaragua	LMIC	22.5	1.8	24.3	27.6	1.0	28.6	+17.7%	74.4%	69.9%	75
Niger	LIC	4.2	5.8	10.0	5.0	3.1	8.1	−19.0%	83.9%	86.9%	441
Nigeria	LMIC	3.2	4.2	7.4	3.8	2.1	5.9	−20.3%	88.1%	90.5%	512
Pakistan	LMIC	9.5	1.2	10.7	11.8	0.6	12.4	+15.9%	88.7%	86.9%	154
Papua New Guinea	LMIC	12.5	3.8	16.3	14.2	2.1	16.3	0.0%	82.8%	82.8%	171
Philippines	LMIC	42.5	1.2	43.7	58.5	0.8	59.3	+35.7%	54.0%	37.6%	78
Rwanda	LIC	24.5	13.2	37.7	28.8	7.8	36.6	−2.9%	39.2%	41.0%	248
Samoa	LMIC	28.5	2.5	31.0	33.2	1.4	34.6	+11.6%	67.4%	63.6%	41
Senegal	LMIC	15.2	4.2	19.4	18.5	2.5	21.0	+8.2%	79.6%	77.9%	243
Sierra Leone	LIC	5.8	6.8	12.6	6.8	3.8	10.6	−15.9%	79.7%	82.9%	717
Somalia	LIC	1.8	3.8	5.6	2.1	1.4	3.5	−37.5%	91.0%	94.4%	692
South Sudan	LIC	2.2	4.2	6.4	2.6	1.8	4.4	−31.3%	89.7%	92.9%	789
Sri Lanka	LMIC	32.5	0.8	33.3	42.5	0.5	43.0	+29.1%	64.9%	54.7%	38
Sudan	LIC	3.5	4.2	7.7	3.9	2.1	6.0	−22.1%	87.6%	90.3%	295
Tanzania	LIC	7.8	6.2	14.0	9.2	3.5	12.7	−9.3%	77.4%	79.5%	238
Timor-Leste	LMIC	28.5	3.2	31.7	35.5	1.8	37.3	+17.7%	66.6%	60.7%	68
Togo	LIC	5.2	5.6	10.8	6.1	3.0	9.1	−15.7%	82.6%	85.3%	392
Uganda	LIC	6.8	5.8	12.6	8.2	3.2	11.4	−9.5%	79.7%	81.6%	284
Ukraine	LMIC	18.5	1.5	20.0	15.2	0.8	16.0	−20.0%	78.9%	83.2%	19
Uzbekistan	LMIC	15.8	1.2	17.0	20.5	0.7	21.2	+24.7%	82.1%	77.7%	25
Vanuatu	LMIC	24.5	2.8	27.3	28.5	1.6	30.1	+10.3%	71.3%	68.3%	68
Vietnam	LMIC	48.5	0.6	49.1	68.5	0.4	68.9	+40.3%	48.3%	27.5%	46
Yemen	LIC	2.5	3.2	5.7	2.8	1.2	4.0	−29.8%	90.8%	93.5%	183
Zambia	LMIC	16.5	4.8	21.3	19.5	3.1	22.6	+6.1%	77.6%	76.2%	278
Zimbabwe	LMIC	14.2	3.8	18.0	16.5	2.2	18.7	+3.9%	81.1%	80.3%	478

Source: Author's projections based on IMF WEO 2025, announced donor cuts with women's health adjustments. Constant 2024 US$ per capita. Gap=(Benchmark-Combined)/Benchmark  ×  100. Benchmark: US$62 LIC, US$95 LMIC. Probabilistic ranges are provided in Appendix G.

MMR, maternal mortality ratio (per 100,000 live births).

## 4 Discussion

### 4.1 Interpretation of findings

The findings of this study provide important evidence regarding the structural determinants of women's health financing and the future trajectory of Universal Health Coverage (UHC) in low-income countries (LICs) and lower-middle-income countries (LMICs). By integrating panel econometric analysis, survival modelling, and spatial analysis, the study demonstrates that women's health financing gaps are not merely temporary fiscal imbalances but represent deeper systemic weaknesses within domestic public finance systems and global development assistance structures. The results reveal three principal findings with significant implications for health financing policy, debt governance, gender-responsive budgeting, and international aid architecture.

#### 4.1.1 Stagnation and marginalization of women's health financing

First, the stagnation of combined government and donor expenditure for women's health services between 2018 and 2025 reflects a systemic failure to prioritize women's health within national and international financing frameworks. Median LIC spending remained approximately US$16 per capita, while median LMIC spending reached only US$45.80 per capita—far below the estimated minimum benchmark required for achieving women-centered UHC targets (4, 5, 26). Moreover, the decline in women's health share within total government health expenditure (GHE), from 18.3% to 15.7%, suggests that women's services are increasingly marginalized during periods of fiscal stress and expenditure compression (7, 8, 12). These findings are highly consistent with feminist political economy literature arguing that fiscal austerity disproportionately affects sectors associated with care work and social reproduction, particularly maternal and reproductive healthcare (7, 12, 13, 32, 38) provide complementary evidence from India showing that fiscal consolidation programmes have disproportionately reduced spending on women's health services, particularly in states with high maternal mortality and weak governance systems, supporting the crowding-out mechanism identified in this study.

The persistence of financing gaps across nearly all LICs and LMICs further indicates that the current trajectory is insufficient for achieving the Sustainable Development Goal (SDG) targets related to maternal health, reproductive health, and gender equality by 2030 (2, 18). WHO estimates documenting stalled reductions in maternal mortality since 2017 support this conclusion, although the present study extends prior evidence by identifying financing stagnation itself as a key explanatory mechanism rather than merely an associated outcome (5, 18). The observed decline in women's health prioritization during periods of macroeconomic stress also aligns with broader evidence demonstrating that governments frequently protect debt obligations and macroeconomic stabilization targets at the expense of social investment (6, 20, 24, 34, 39) further demonstrate that structural inequalities in health financing are associated with widening disparities in maternal health outcomes, with the poorest women in aid-dependent countries experiencing the greatest reductions in service utilization when donor funding declines.

Importantly, the COVID-19 period appears to have intensified gendered financing inequalities. Countries experiencing the largest pandemic-related debt increases also demonstrated the sharpest declines in women's health expenditure shares. This finding reflects broader global concerns that the pandemic reversed progress in women's access to reproductive, maternal, and sexual health services (28–31). International evidence from family planning and HIV financing also indicates that women-focused programmes remain especially vulnerable to expenditure cuts because of their dependence on external funding and their relatively weak political visibility within domestic budgeting systems (29–31, 40) extend this analysis to Sub-Saharan Africa through their global health trajectories work, showing that debt distress is associated with higher maternal mortality ratios and lower skilled birth attendance, with the effects being most pronounced in countries with weak governance systems and high aid dependency.

#### 4.1.2 Debt service crowding-out: A gendered fiscal phenomenon

Second, the statistically significant crowding-out effect of debt service on women's health expenditure (*β* = −0.042, *p* < 0.001) provides one of the clearest quantitative demonstrations of how debt distress constrains gender-responsive health financing. Critically, this effect is substantially larger than the crowding-out effect on general health expenditure (*β* = −0.012, *p* = 0.032), with the difference being statistically significant (*p* = 0.008). This finding—that debt service crowds out women's health spending 3.5 times more strongly than general health spending—represents the study's headline result and reveals the gendered distributional consequences of fiscal adjustment. Existing fiscal space literature has long argued that rising debt obligations reduce governments’ ability to expand social expenditure (20, 24, 34), yet relatively few studies have specifically examined the gendered distributional consequences of debt servicing. The present findings demonstrate that women's health spending is even more vulnerable to fiscal compression than general health expenditure, suggesting that women's services are frequently treated as residual expenditure categories during fiscal adjustment periods (7, 32, 41) further demonstrate that debt service burdens in South Asian countries are associated with reduced government spending on nutrition and maternal health programmes, with the poorest and most marginalized women bearing the greatest burden of these fiscal adjustments.

This result has major implications for ongoing global debates surrounding sovereign debt restructuring and fiscal sustainability in developing economies. Median LICs now allocate larger shares of public expenditure to interest payments than to public health systems, while women's health receives only a fraction of total government expenditure (6, 20). Consequently, debt servicing directly competes with investments in maternal health, sexual and reproductive healthcare, and primary healthcare systems. The findings therefore support calls from global health advocates and feminist economists for debt restructuring frameworks that explicitly incorporate social expenditure protections, particularly for women's health services (7, 12, 32).

The estimated potential gains from debt-for-health swaps are particularly important. Simulations suggest that targeted debt relief could mobilize billions of dollars annually for maternal and reproductive health programmes across debt-distressed LICs and LMICs. Such findings reinforce broader arguments that investments in women's health should be viewed not as consumption expenditure but as long-term human capital investments generating substantial economic returns through increased productivity, labour participation, and intergenerational welfare improvements (1–3, 33). Theoretical frameworks developed by Musgrave, Wagner, and Rostow similarly suggest that public expenditure on social sectors expands alongside economic modernization and state development (35–37). However, the current findings indicate that debt distress can interrupt this developmental trajectory by constraining fiscal expansion precisely when health investment needs are greatest.

The spatial clustering of financing declines within Sub-Saharan Africa further highlights the importance of regional political economy dynamics. Countries within the Nigeria–Cameroon–Chad–Niger–CAR corridor exhibited especially severe declines in women's health financing despite simultaneously experiencing some of the world's highest maternal mortality burdens (18). This clustering likely reflects shared structural vulnerabilities, including commodity dependence, external debt exposure, weak domestic revenue systems, donor dependency, and fragile health infrastructure (19, 20, 28). The findings therefore imply that regional financing mechanisms, pooled procurement systems, and cross-border maternal health initiatives may prove more effective than isolated national interventions in addressing women's health financing gaps.

#### 4.1.3 Development assistance reductions and UHC Gap closure

Third, the Cox proportional hazards analysis demonstrates that reductions in development assistance for health (DAH) substantially reduce the probability of achieving women's UHC financing thresholds by 2030. The estimated hazard ratio of 0.939 per percentage-point decline in aid indicates that countries experiencing major donor withdrawals face dramatically reduced probabilities of financing gap closure. This finding is especially relevant in the context of recent reductions in external assistance from major bilateral donors and multilateral financing agencies (9–11). Women's health programmes—including maternal healthcare, HIV/AIDS services, and family planning initiatives—remain highly dependent on external financing in many LICs (21, 28–31). Consequently, abrupt aid reductions create immediate financing shocks that domestic fiscal systems are often unable to absorb (42) show through their health systems strengthening analysis that aid-dependent countries with weak governance systems and high debt burdens are least able to compensate for donor withdrawal, creating a “financing trap” that perpetuates underinvestment in women's health.

The findings challenge optimistic assumptions that domestic resource mobilization can rapidly substitute for declining aid flows. Although domestic financing expansion remains essential for long-term sustainability (14, 24, 25), many LICs continue to face structural constraints, including narrow tax bases, weak administrative capacity, high debt obligations, and competing expenditure priorities (6, 20, 34). These constraints are compounded by gendered political economy dynamics in which women remain underrepresented within fiscal decision-making institutions and budget allocation processes (12, 13, 17). Therefore, rapid aid withdrawal risks entrenching long-term underinvestment in women's health systems (43). demonstrate through their maternal health financing work that phased aid transitions and demand-side financial interventions are associated with successful domestic resource mobilization and sustained service coverage, providing a model for other aid-dependent countries.

At the same time, the study identifies gender-responsive budgeting as an important institutional mechanism capable of mitigating financing vulnerabilities. Countries implementing stronger gender budgeting systems demonstrated stronger positive relationships between domestic expenditure and women's health financing outcomes. This finding extends previous descriptive studies on gender budgeting by providing rigorous quantitative evidence linking institutionalized gender-responsive budgeting to measurable expenditure improvements (13, 17). The estimated coefficient associated with gender budgeting suggests that improvements in institutional gender budgeting frameworks could produce expenditure gains comparable to substantial increases in GDP per capita. This reinforces arguments from feminist public finance scholars that gender budgeting should be treated not merely as an administrative reform but as a core mechanism for improving equity and allocative efficiency within health systems (12, 13, 16, 17).

### 4.2 Comparison with previous literature

Several findings differ from earlier studies on health financing elasticity and aid dependence. The estimated GDP elasticity of women's health expenditure (0.398) is considerably lower than the near-unitary elasticities previously reported for aggregate health spending (14, 15) and also lower than estimates from prior gender-focused analyses (16). One likely explanation is that the present study captures the post-COVID fiscal environment characterized by heightened debt burdens, expenditure compression, and declining aid flows (6, 11, 20, 28). Earlier studies conducted during periods of stronger global economic growth may therefore have overestimated governments’ capacity to translate economic growth into sustained health expenditure expansion.

Methodological differences may also explain variation across studies. Unlike several earlier analyses relying primarily on National Health Accounts or aggregate expenditure estimates, the present study incorporates executed budget data and women-specific expenditure tracking mechanisms (5, 8). This approach more accurately captures real fiscal allocation dynamics and gender-specific expenditure priorities. The inclusion of debt service variables and gender budgeting indicators additionally provides a more comprehensive framework for understanding the determinants of women's health financing trajectories.

The study's findings regarding development assistance similarly contribute to ongoing debates within the aid effectiveness literature. The positive but relatively weak coefficient associated with off-budget DAHW differs from earlier evidence documenting substantial aid displacement effects in some African countries (22). However, the results align more closely with recent studies suggesting that aid effects vary according to institutional quality, governance systems, and fiscal management capacity (21, 23). The subgroup findings showing stronger crowding-in effects within countries implementing gender budgeting frameworks further suggest that institutional context significantly shapes how external assistance influences domestic expenditure decisions.

Overall, the findings contribute to a growing body of literature emphasizing that achieving UHC for women's health requires more than aggregate expenditure growth alone. Sustainable progress depends on gender-responsive fiscal institutions, protected social expenditure during debt crises, stable development assistance transitions, and deliberate political prioritization of women's health within public finance systems (12–17, 24–27). Without such reforms, the financing trajectories observed during the 2018–2025 period is likely to persist, placing the 2030 SDG targets for women's health increasingly out of reach for many LICs and LMICs.

### 4.3 Policy implications

The empirical findings support five principal policy recommendations for strengthening women’s health financing.

- Protect women's health budgets from debt-service crowding out through debt-for-health swaps. For the 28 LICs and LMICs currently experiencing or at high risk of debt distress, the international community should prioritize debt-for-health swaps specifically targeting maternal health and family planning services. Recent examples, including debt-for-health swaps implemented in Côte d'Ivoire and Belize, demonstrate feasibility. The 3.5-fold stronger crowding-out effect on women's health compared to general health provides a compelling rationale for gender-targeted debt relief.
- Front-load domestic resource mobilization for women's health in countries with fiscal space. Among the 23 countries classified as fiscal expansion countries, a one percentage point increase in women's health share of government expenditure could substantially increase per capita women's health spending. Governments should adopt women's health spending floor legislation.
- Manage aid transitions over longer time horizons (5–10 years) rather than abrupt withdrawal. Development partners should adopt predictable multi-year transition frameworks with annual reduction caps of no more than 10%-15% per year. The hazard ratio of 0.939 per percentage-point aid decline underscores the severe consequences of rapid aid withdrawal.
- Strengthen gender-responsive budgeting and expenditure tracking systems. Governments should adopt sex-disaggregated health accounts, implement gender budget statements within the health sector, and require civil society participation in budget monitoring. The strong positive coefficient for gender budgeting (*β* = 0.024, *p* < 0.001) provides empirical support for these institutional reforms.
- Reallocate within health budgets toward primary healthcare for women. The median PHC share of women's health spending is only 18%, far below the recommended 30%-40% target. Countries should conduct gender-sensitive public expenditure reviews to identify inefficient spending and redirect resources toward cost-effective primary care services for women.

### 4.4 Limitations

Eight limitations should be acknowledged. (1) Nowcasting assumes historical budget execution patterns, potentially introducing measurement error in countries experiencing macroeconomic instability or political conflict. (2) The absence of standardized sex-disaggregated health accounts in many countries may lead to residual measurement error that attenuates estimated coefficients. (3) Projections assume constant women's health shares through 2030 under baseline scenarios, which may under- or over-estimate future growth. (4) DAH reductions are based on announced cut point estimates; actual reductions may vary, and probabilistic ranges in Appendix G should be considered. (5) UHC costing benchmarks are global averages and may not fully reflect country-specific circumstances. (6) The 80% financing benchmark threshold for gap closure is supported by WHO guidance but remains a simplifying assumption. (7) Patient-level validation is correlational, not causal; bootstrap-corrected standard errors address first-stage uncertainty but not unobserved confounding. (8) The study focuses primarily on financing rather than all health outcomes; maternal mortality was included as a validation measure, but the analysis does not directly estimate relationships between financing trajectories and all women's health outcomes.

#### 4.4.1 Projection uncertainty

The projections presented in this study should be interpreted with appropriate caution. The point estimates in Tables 16, 5 represent modal outcomes from the Monte Carlo simulation, but substantial uncertainty surrounds these estimates. The probabilistic scenario ranges provided in Appendix G indicate that the 31.3% LIC closure probability under the combined reform scenario ranges from 22% to 41% depending on assumptions about aid cuts, GDP growth, and domestic fiscal responses. The discussion should therefore be read as identifying plausible trajectories rather than deterministic predictions.

## 5 Conclusion

Low- and lower-middle-income countries stand at a critical crossroads. This study demonstrates that progress has stalled, with no LICs and few LMICs on track to meet women's UHC financing benchmarks by 2030. Debt service crowds out women's health spending 3.5 times more strongly than general health spending (*β* = −0.042 vs. −0.012, *p* for difference=0.008), and announced donor cuts make gap closure nearly impossible without major domestic reprioritization. However, combined reform scenarios integrating modest expenditure share increases (≥2.5 percentage points), managed aid transitions, primary healthcare reallocation, and strengthened gender budgeting could raise LIC closure probability from 0% to 31% (range: 22%–41% under probabilistic scenarios).

The central issue is not whether the financing gap can be closed, but whether national governments and the global community possess the political will to act with the urgency that women's health demands. The consequences of inaction are measured in preventable maternal deaths, unmet need for reproductive health services, avoidable cervical cancer mortality, and the perpetuation of intergenerational cycles of poverty and ill health. The evidence suggests that the time for decisive action is now, before aid reductions intensify and debt service further erodes the limited fiscal space available for women's health. As the global community approaches the 2030 SDG deadline, the imperative for action on women's health financing has never been more urgent.

## Data Availability

The original contributions presented in the study are included in the article/Supplementary Material, further inquiries can be directed to the corresponding author.
