# Supplementary material for: Why women’s health financing collapses when donors retreat and interest payments rise
Source: Front Glob Womens Health. 2026 Oct 21;7:1894105. doi: 10.3389/fgwh.2026.1894105 (PMC13731282; doi:10.3389/fgwh.2026.1894105)
Supplement: Supplementary file 1 [file Datasheet1.pdf]

APPENDICES

Appendix A: Sensitivity Analysis Excluding Countries with >50% Nowcasted Data (2024-2025)

To assess the robustness of findings to nowcasting assumptions, Model 1 was re-estimated excluding country-years with >50% nowcasted data (2024-2025, n=44 countries).

Table A.1: Sensitivity Analysis - Excluding Countries with >50% Nowcasted Data

Variable	Coefficient (Full Sample)	Coefficient (Excluding >50% Nowcasted)	Difference
Lagged GHEW (log)	0.671*** (0.079)	0.648*** (0.084)	-0.023
GDP per capita (log)	0.398*** (0.107)	0.412*** (0.112)	+0.014
Debt service (% GDP)	-0.042*** (0.010)	-0.038*** (0.012)	+0.004
Off-budget DAHW (log)	0.088* (0.046)	0.092* (0.049)	+0.004
Health share of GGE (%)	0.139*** (0.030)	0.134*** (0.032)	-0.005
Gender budgeting index	0.024*** (0.007)	0.022*** (0.008)	-0.002
Polity score	0.015* (0.008)	0.014* (0.008)	-0.001
Observations	497	369	—
R-squared (within)	0.781	0.769	—
First-stage F-statistic	41.2	38.7	—

Source: Author's calls. Notes: ***$p<0.01$, **$p<0.05$, _$p<0.1$. Robust SE in parentheses. Full sample n=497; restricted sample n=369 (excludes 2024-2025 observations from countries with >50% nowcasted data)._

Interpretation: The debt service coefficient remained negative and significant ($\beta = -0.038$, $p = 0.002$) when excluding countries with >50% nowcasted data, comparable to the main estimate ($\beta = -0.042$, $p < 0.001$). The gender budgeting coefficient remained positive and significant ($\beta = 0.022$, $p = 0.004$). These results indicate that findings are not driven by nowcasting procedures.

Appendix B: Robustness Checks with Lagged Debt Service

To address concerns about endogeneity of debt service, Model 1 was re-estimated using lagged debt service (t-1) and debt service averaged over 3 years.

Table B.1: Robustness Checks - Alternative Debt Service Specifications

Specification	Debt Service Coefficient	SE	p-value	R^2	N
Main (contemporaneous)	-0.042***	0.010	<0.001	0.781	497
Lagged (t-1) debt service	-0.039***	0.011	<0.001	0.774	426
3-year average debt service	-0.041***	0.012	0.001	0.776	426
Debt service excluding interest	-0.035***	0.013	0.007	0.768	497

Source: Author's calculations. Notes: **_$p<0.01$. All models include same covariates as Model 1. Robust SE clustered by country._

Interpretation: The debt service crowding-out effect remained negative and statistically significant across all alternative specifications, with coefficients ranging from -0.035 to -0.042. This provides confidence that the main finding is not driven by the specific timing of the debt service variable.

Appendix C: Additional Instrument Diagnostics

Detailed instrument diagnostics for the instrumental variables' strategy employed in Model 1.

Table C.1: Instrument Diagnostics - First Stage Results

**Instrument**	**Coefficient on DAHW (log)**	**SE**	**F-statistic**	**Partial R^2**
Donor political alignment (UN voting)	0.342***	0.079	18.7	0.238
Historical aid allocation (lagged t-2)	0.286***	0.076	14.2	0.201
Donor budget cycles	0.184**	0.064	8.3	0.134
**All instruments combined**	—	—	**41.2**	**0.412**

Source: Author's calculations. Notes: ***p<0.01, *p<0.05. *First-stage regression includes all Model 1 covariates. N=497.*

Table C.2: Overidentification and Weak Instrument Tests

**Test**	**Statistic**	**p-value**	**Critical Value**	**Conclusion**
Hansen J	2.14	0.342	χ^2 (2) = 5.99 (5%)	Cannot reject null (instruments valid)
Cragg-Donald Wald F	41.2	<0.001	10.0 (Stock-Yogo)	Reject weak instruments
Angrist-Pischke F (donor alignment)	18.7	<0.001	10.0	Strong instrument
Angrist-Pischke F (historical)	14.2	<0.001	10.0	Strong instrument
Angrist-Pischke F (budget cycles)	8.3	0.004	10.0	Moderate instrument
Durbin-Wu-Hausman	8.94	0.011	χ^2 (1) = 3.84 (5%)	Reject exogeneity (IV needed)

Source: Author's calculations.

Interpretation: The instruments are strong (first-stage F=41.2 > 10), valid (Hansen J non-significant), and the endogeneity test confirms that IV estimation is necessary. The Angrist-Pischke F-statistics indicate that donor alignment (18.7) and historical allocation (14.2) are strong instruments, while budget cycles (8.3) are moderately strong and are retained for overidentification testing.

Appendix D: Alternative Survival Model Specifications

To assess robustness of the survival analysis results, four alternative specifications were estimated.

Table D.1: Alternative Survival Model Specifications - Hazard Ratios

Variable	Model 2 (Cox)	Model 2a (Weibull)	Model 2b (AFT - Log-normal)	Model 2c (Discrete-time Logit)	Model 2d (Competing Risks)
GHEW growth	1.198**	1.185**	1.192**	1.201**	1.194**
Aid decline	0.939***	0.942***	0.938***	0.941***	0.940***
Fiscal space	2.947**	2.834**	2.956**	2.901**	2.912**
PHC women's share	1.896*	1.878*	1.902*	1.884*	1.891*
Gender budgeting	1.042**	1.040**	1.043**	1.041**	1.042**
Governance	1.458	1.442	1.461	1.450	1.455
MMR baseline	0.972	0.968	0.974	0.970	0.971
Debt baseline	0.945	0.948	0.943	0.946	0.944
**AIC**	**412.8**	**414.2**	**413.5**	**415.1**	**416.0**

Source: Author's calculations. Notes: *p<0.10, **p<0.05, **_p<0.01. AIC = Akaike Information Criterion. Model 2d treats "gap closure" and "aid cessation" as competing events._

Interpretation: The hazard ratios remained consistent across all alternative specifications. The Cox model (Model 2) had the lowest AIC (412.8), indicating the best fit. The competing risks model (Model 2d) produced similar results, suggesting that the main findings are not sensitive to the specific survival model chosen.

Appendix E: Spatial Sensitivity Analyses

E.1: Sensitivity to Spatial Weight Threshold (300km and 700km)

Table E.1: Global Moran's I - Sensitivity to Threshold Distance

Indicator	500km (Main)	300km	700km
GHEW per capita (2024)	0.324***	0.298**	0.335***
Change in GHEW (2024-2030)	0.358***	0.321**	0.369***
Women's health financing gap (2024)	0.298**	0.272**	0.310**
Maternal mortality ratio (2024)	0.442***	0.418***	0.451***

Source: Author's calculations. Notes: **p<0.05, **_p<0.01. Inverse distance weights. 999 permutations._

Interpretation: The spatial autocorrelation results remained consistent across all threshold distances (300km, 500km, and 700km), confirming that the findings of moderate positive spatial clustering are robust to the choice of spatial weight threshold.

E.2: LISA Cluster Composition

Table E.2: LISA Cluster Composition by Country

Cluster Type	Countries	Number
**High-High (decline)**	Nigeria, Cameroon, Chad, Niger, Central African Republic	5
**Low-Low (growth)**	Kenya, Tanzania, Uganda, Rwanda, Burundi	5

**High-Low**	Ethiopia, Somalia	2
**Low-High**	Ghana, Côte d'Ivoire	2
**Not significant**	Remaining 57 countries	57

Source: Author's calculations. Notes: High-High cluster indicates countries with high decline in GHEW surrounded by countries with high decline. Low-Low cluster indicates countries with low decline (growth) surrounded by countries with low decline (growth). Thailand was included in the descriptive map for regional context but excluded from the formal cluster analysis as it is outside the 71-country income-group sample.

Appendix F: Gender Budgeting Index - Detailed Methodology

The gender budgeting index ranges from 0-100 and is derived from the World Bank Gender Data Portal indicators measuring four dimensions of gender-responsive budgeting in the health sector.

Table F.1: Gender Budgeting Index Components and Scoring

Dimension	Indicator	Data Source	Scoring (0-25 per dimension)
**1. Institutional Framework**	Existence of gender-responsive budgeting framework in health sector	World Bank Gender Data Portal / UN Women	0 = No framework; 8 = Framework under development; 17 = Framework adopted; 25 = Framework fully implemented
**2. Expenditure Tracking**	Implementation of sex-disaggregated health accounts	WHO GHED / World Bank	0 = No sex-disaggregated accounts; 8 = Pilot stage; 17 = Partial implementation; 25 = Fully implemented
**3. Parliamentary Oversight**	Parliamentary oversight of gender budgeting in health	Inter-Parliamentary Union / World Bank	0 = No oversight; 8 = Limited oversight; 17 = Regular oversight; 25 = Strong oversight with enforcement
**4. Civil Society Participation**	Civil society participation in budget monitoring	World Bank / UNDP	0 = No participation; 8 = Limited consultation; 17 = Regular consultation; 25 = Active participation with influence

Source: Author's compilation based on World Bank Gender Data Portal (2025), UN Women (2020), and WHO GHED (2024).

Table F.2: Validation of Gender Budgeting Index

Validation Method	Result	p-value
Correlation with UN Women gender budgeting assessments	r = 0.87	<0.001
Correlation with IMF gender budgeting scores	r = 0.82	<0.001
Cronbach's alpha (internal consistency)	α = 0.89	—
Inter-rater reliability (across coders)	ICC = 0.91	<0.001

Source: Author's calculations.

Interpretation: The gender budgeting index demonstrates strong validity (r=0.87 with UN Women assessments), high internal consistency (α=0.89), and excellent inter-rater reliability (ICC=0.91). Countries scoring 0-33 were classified as "no gender budgeting," 34-66 as "partial gender budgeting," and 67-100 as "comprehensive gender budgeting."

Appendix G: Probabilistic Scenario Ranges for 2030 Projections

This appendix presents the probabilistic ranges around the main projection estimates, generated using Monte Carlo simulation (1,000 draws) incorporating uncertainty around:

1. Donor cut magnitudes (US: 50-70%; UK: 35-45%; France: 20-30%; Germany: 5-15%)
2. GDP growth rates (±1.5 percentage points around IMF projections)
3. Women's health share changes (±1 percentage point around assumed trajectories)
4. Debt service trajectories (±2 percentage points around baseline)

Table G.1: Predicted Women's UHC Gap Closure Probabilities by 2030 - Probabilistic Ranges

Scenario	LICs (n=32)	LMICs (n=39)	Total (n=71)
**Baseline**			
Mean	0.0%	23.1%	12.7%
5th-95th percentile	0.0-0.0%	18.2-28.5%	9.2-16.3%
**Optimistic GHE growth (+2.5pp)**			
Mean	12.5%	43.6%	29.6%
5th-95th percentile	8.4-17.2%	37.4-50.1%	24.0-35.4%
**Moderate aid restoration (50%)**			
Mean	3.1%	30.8%	18.3%
5th-95th percentile	1.2-5.8%	25.2-36.7%	14.2-22.8%
**PHC efficiency**			
Mean	0.0%	28.2%	15.5%
5th-95th percentile	0.0-0.0%	22.8-34.0%	11.9-19.4%
**Gender budgeting (+20 points)**			
Mean	0.0%	33.3%	18.3%
5th-95th percentile	0.0-0.0%	27.6-39.4%	14.6-22.4%
**Combined reform**			
Mean	31.3%	66.7%	50.7%
5th-95th percentile	22.1-41.2%	58.4-75.3%	42.8-59.1%
**Full fiscal space utilization**			
Mean	37.5%	79.5%	60.6%
5th-95th percentile	28.3-47.4%	71.2-88.1%	52.4-69.2%

Source: Author's projections based on Model 2 hazard ratios and Monte Carlo simulation (1,000 draws).

Table G.2: Combined Women's Health Spending (US$ per capita) - Probabilistic Ranges by 2030

Country Group	2024 Mean	2030 Baseline Mean	2030 Baseline (5th-95th)	2030 Combined Reform Mean	2030 Combined Reform (5th-95th)
**LICs**					
Median	16.25	16.40	14.85-17.95	22.40	19.85-24.95
Mean	18.35	18.12	16.42-19.82	24.58	21.78-27.38
**LMICs**					
Median	44.60	45.80	42.10-49.50	58.40	53.80-63.00
Mean	47.82	48.45	44.55-52.35	61.78	56.92-66.64

Source: Author's projections based on Monte Carlo simulation (1,000 draws).

Interpretation: The probabilistic ranges indicate substantial uncertainty around the point estimates. For the combined reform scenario, the LIC closure probability ranges from 22% to 41% (5th-95th percentile), meaning there is a 90% probability that the true closure rate falls within this range. The median LIC combined spending under the combined reform scenario ranges from US$19.85 to US$24.95, compared to US$14.85 to US$17.95 under baseline.

Appendix H: Data Sources and Variable Definitions

Table H.1: Data Sources and Variable Definitions

Variable	Definition	Source	Years Available	Coverage
GHEW	Government health expenditure for women's health services per capita (2024 US$)	WHO GHED + budget documents	2018-2025	71 countries
DAHW	Off-budget development assistance for women's health per capita (2024 US$)	OECD CRS + IHME DAH	2018-2025	71 countries
GDP per capita	Gross domestic product per capita (2024 US$)	IMF WEO	2018-2025	71 countries
Debt service	Interest payments as % of GDP	IMF WEO	2018-2025	71 countries
Tax revenue	Tax revenue as % of GDP	IMF WEO	2018-2025	71 countries
Health share of GGE	Health expenditure as % of general government expenditure	WHO GHED	2018-2025	71 countries
Gender budgeting index	Composite index of gender-responsive budgeting (0-100)	World Bank Gender Data Portal	2018-2025	71 countries
Polity score	Democracy score (-10 to 10)	Polity V	2018-2025	71 countries
Governance indicator	Average of 6 governance dimensions	World Bank WGI	2018-2025	71 countries
Maternal mortality ratio	Maternal deaths per 100,000 live births	WHO GHO	2018-2025	71 countries
Skilled birth attendance	% of births attended by skilled health personnel	WHO GHO	2018-2025	71 countries
Modern contraceptive prevalence	% of women using modern contraception	WHO GHO	2018-2025	71 countries
PHC women's share	% of GHEW allocated to primary healthcare for women	WHO GHED	2018-2025	71 countries

Appendix I: Country Income Classification

Table I.1: Country Income Classification (World Bank, 2024)

Income Group	Countries (n=71)
**LICs (n=32)**	Afghanistan, Benin, Burkina Faso, Burundi, Central African Republic, Chad, Congo Dem. Rep., Eritrea, Ethiopia, Gambia, Guinea, Guinea-Bissau, Haiti, Liberia, Madagascar, Malawi, Mali, Mozambique, Niger, Rwanda, Sierra Leone, Somalia, South Sudan, Sudan, Tanzania, Togo, Uganda, Yemen, plus 4 others
**LMICs (n=39)**	Angola, Bangladesh, Bhutan, Bolivia, Cabo Verde, Cambodia, Cameroon, Comoros, Congo Rep., Côte d'Ivoire, Djibouti, Egypt, El Salvador, Eswatini, Ghana,

	Honduras, India, Indonesia, Kenya, Kiribati, Kyrgyzstan, Lao PDR, Lesotho, Marshall Islands, Mauritania, Micronesia, Mongolia, Morocco, Myanmar, Namibia, Nepal, Nicaragua, Nigeria, Pakistan, Papua New Guinea, Philippines, Samoa, São Tomé and Príncipe, Senegal, Solomon Islands, Sri Lanka, Timor-Leste, Tuvalu, Ukraine, Uzbekistan, Vanuatu, Vietnam, Zambia, Zimbabwe

Source: World Bank country classification, 2024 fiscal year.
